# The Future of Neuroscience: Flexible and Wireless Implantable Neural Electronics

**DOI:** 10.1002/advs.202002693

**Published:** 2021-03-09

**Authors:** Eve McGlynn, Vahid Nabaei, Elisa Ren, Gabriel Galeote‐Checa, Rupam Das, Giulia Curia, Hadi Heidari

**Affiliations:** ^1^ Microelectronics Lab James Watt School of Engineering University of Glasgow Glasgow G12 8QQ United Kingdom; ^2^ Laboratory of Experimental Electroencephalography and Neurophysiology Department of Biomedical Metabolic and Neural Sciences University of Modena and Reggio Emilia Modena 41125 Italy

**Keywords:** biocompatible encapsulation, brain implantable device, neural interface, neural probe design, wireless data transfer, wireless power

## Abstract

Neurological diseases are a prevalent cause of global mortality and are of growing concern when considering an ageing global population. Traditional treatments are accompanied by serious side effects including repeated treatment sessions, invasive surgeries, or infections. For example, in the case of deep brain stimulation, large, stiff, and battery powered neural probes recruit thousands of neurons with each pulse, and can invoke a vigorous immune response. This paper presents challenges in engineering and neuroscience in developing miniaturized and biointegrated alternatives, in the form of microelectrode probes. Progress in design and topology of neural implants has shifted the goal post toward highly specific recording and stimulation, targeting small groups of neurons and reducing the foreign body response with biomimetic design principles. Implantable device design recommendations, fabrication techniques, and clinical evaluation of the impact flexible, integrated probes will have on the treatment of neurological disorders are provided in this report. The choice of biocompatible material dictates fabrication techniques as novel methods reduce the complexity of manufacture. Wireless power, the final hurdle to truly implantable neural interfaces, is discussed. These aspects are the driving force behind continued research: significant breakthroughs in any one of these areas will revolutionize the treatment of neurological disorders.

## Graphical abstract

In this progress report, the evolution of neural stimulation is explored with a view to providing practical design guidelines for innovative, flexible, and fully integrated implantable probes. From the foreign body response, to the material characteristics of the brain, all aspects of the target tissue must be accommodated. Fabrication techniques, as well as wireless power approaches, are also discussed.

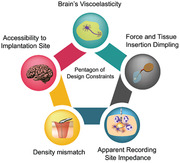

## 1 Introduction

Neural interface recording and stimulation has been demonstrated as an effective diagnosis and treatment for numerous neurological disorders.^[^ ^1^ ^]^ With an emphasis on building closed‐loop systems, modern neural probes must incorporate both these functionalities while addressing the disparate design requirements for different types of electrodes, perhaps going so far as to incorporate different modalities for each task. As such, rapid growth has occurred in this area of research, with several distinctive and promising advances in neural probe design.^[^ ^2^ ^]^ Bioelectronic medicine is a fast‐growing field, which seeks to establish new brain stimulation techniques, while achieving two important goals: first, to reduce negative side effects on the patient, and secondly, to mitigate technical problems associated with the current designs available for brain implants. As these technologies have become more sophisticated, the number of applications for neural stimulation have increased, now encompassing a wide range of neurological and mood disorders, from Parkinson's disease (PD) to depression. Although the number of deaths from disorders such as epilepsy and PD are declining, a concerted effort is required to improve patients’ quality of life, especially when measuring disability‐adjusted life‐years.^[^ ^3^ ^]^ Innovative neurotechnologies focusing on the recording and stimulus of brain activity could alleviate the burden on sufferers and their caregivers. Recent emphasis on flexible, miniaturized neural probes has shaped the latest generation of implant design. Only through optimizing each step in the process of probe design and fabrication, robust and innovative neural probes will be created, and carried into the future of patient care.

In this progress report, the history of neural implants is explored, including the most recent and successful approaches, and how they evolved from early technologies. The practicalities of the neural probe, namely the shape and scale, have seen excellent progress in recent years with the advent of tissue‐like materials and mesh electronics. In order to prevent an immune response, the probes must mimic the flexibility, softness, and micron‐scale features of target organs/tissues. However, these characteristics present a challenge in terms of implantation: a compromise must be found between flexibility and stiffness during surgery in order to minimize unnecessary tissue damage.^[^ ^4^ ^]^ A fully flexible probe requires a potentially large area of the skull and dura mater to be removed, which will hamper the healing process. Although probe design has improved far beyond traditional silicon, wired probes will always be limited, and prevented from integrating seamlessly with the tissue.^[^ ^5^ ^]^ Wireless, batteryless power represents a bottleneck for chronic implantation, and progression to the clinic.

## 2 Stimulation Methods

The emergence of neural stimulation dates from 46 C.E., when electric ray fish were used to treat head pain,^[^ ^6^ ^]^ while the 1950s saw the first development of electroconvulsive therapies for some neurological disorders. At the same time, researchers were starting to use deep brain stimulation (DBS) techniques in animal research, with the goal of electrically stimulating a number of different parts of the brain. Satisfactory results in several areas, including behavioral effects were found and thus, paved the way for these techniques to be used on human participants.^[^ ^7^, ^8^, ^9^ ^]^ Since then, DBS has been a useful stimulation technique for numerous neurological disorders.^[^ ^1^ ^]^ However, unspecific electric stimulation of the brain may obscure the true result of stimulating the target tissue,^[^ ^10^ ^]^ or have other severe complications that might be overcome through the use of novel stimulation methods. The use of these novel technologies could improve efficiency and reduce negative effects of brain electrical stimulation. In the last decade, optogenetic technology has emerged as a new opportunity to solve the issues associated with electric stimulation. However, it is still in development and not commercialized for human use. In this section, the main technical aspects of those stimulation methods are described, indicating why some techniques have fallen out of favor with clinicians.

**Figure 1:**
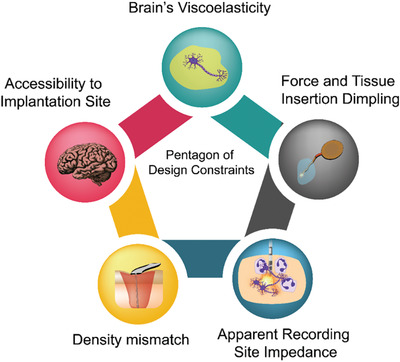
Pentagon of design constraints of brain implants. Accessibility to implantation site, density mismatch, apparent recording site impedance, force and tissue insertion dimpling, and the brain tissue viscoelasticity are the main issues to overcome during the design of brain implantable device in chronic implantation.^[^ ^11^ ^]^

Among the numerous obstacles shaping the design of neural implants, the most important are: difficult accessibility to the implantation site; viscoelastic properties of the brain; tissue dimpling and scattering due to the injection force during probe implantation; density mismatch between the probe material and brain tissue and the physiologic fluid; and apparent recording site impedance.^[^ ^11^, ^12^, ^13^ ^]^ Those five restrictions are compiled in **Figure** **1**. Material choice and electronic design are two factors that will determine the biocompatibility and long‐term viability of the implantable device which are dictated by the stimulation technique.^[^ ^11^, ^12^ ^]^ More precisely, mechanical properties are the main concern of most research in this field.

**Figure 2:**
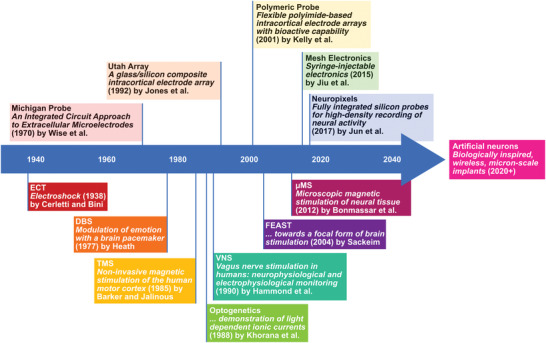
Significant publications in the field of neural stimulation, which illustrate the place of electrically stimulating probes amongst the development of alternative techniques. The upper section includes such important designs as: the Michigan probe,^[^ ^360^ ^]^ the Utah Array,^[^ ^361^ ^]^ Polymeric probes,^[^ ^362^ ^]^ Mesh Electronics,^[^ ^296^ ^]^ and Neuropixels.^[^ ^140^ ^]^ The lower half of the timeline includes Electroconvulsive Therapy,^[^ ^363^ ^]^ Deep Brain Stimulation,^[^ ^364^ ^]^ Transcranial Magnetic Stimulation,^[^ ^365^ ^]^ Optogenetics,^[^ ^366^ ^]^ Vagus nerve stimulation (VNS),^[^ ^367^ ^]^ FEAST,^[^ ^21^ ^]^ and μMS.^[^ ^33^ ^]^

Neural stimulation probes typically incorporate a conductive material, such as gold.^[^ ^14^ ^]^ Although it is an inert material, and its toxicity is very low to the body, ensuring a biochemical compatibility with the brain tissue,^[^ ^15^ ^]^ the reality is that in mechanical terms, size matters. This highly determines the long‐term viability of the implant functionality. Using these data presented here and given the design constraints of this implant typology, several conventional therapies will be introduced, as well as the progress on the designs trying to overcome those restrictions. These therapies range from the oldest and least common technique, such as electroshock, to the most novel therapy in development, such as microscopic magnetic stimulation (μMS). Breakthroughs in the various electronic, magnetic, and optical stimulation techniques which have evolved over the decades are summarized in **Figure** **2**.

While a range of techniques have been developed over the past decade to modulate and record neural activity, Figure 2 details how miniaturized electrical probes have developed alongside alternative methods. Despite the benefits of emerging technologies, significant effort is still expended by the research community to improve the design of electrical probes. In recent years, especially, major strides have been taken towards fully implantable chronic recording probes, with a view to creating closed‐loop systems which do not recruit an immune response in the future.

### 2.1 Electroconvulsive Therapy

**Figure 3:**
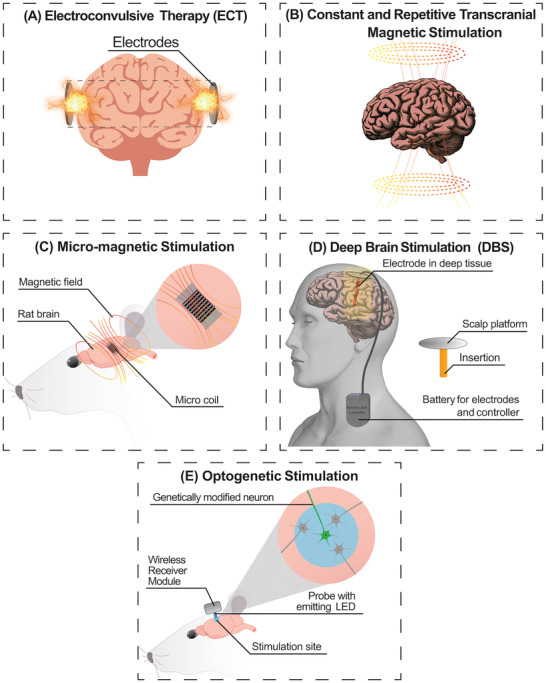
Summary of the four main brain stimulation techniques based on electrical systems. A) Electroconvulsive Therapy is the most conventional technique, which was replaced by B) Transcranial Magnetic Stimulation for some specific cases of neurological disorders. C) Micromagnetic stimulation requires the implantation of a miniaturized magnetic coil which will not be hindered by immune cell encapsulation. D) Deep Brain Stimulation systems are the most used like commercial products, e.g., Neuropace, Medtronic VNS, and so on. E) Optogenetic stimulation has appeared as an innovative and interesting opportunity during the last decade with the use of Chr2.

During the 1940s, a technique called electroconvulsive therapy (ECT; **Figure** **3**A) appeared as a novel intervention method for the treatment of major depressive disorders and severe mood illnesses.^[^ ^16^ ^]^ During ECT, also known as shock therapy, a small electric current is delivered through electrodes into the brain triggering a brief seizure and neurochemical changes. It was considered very effective in terms of time and resources, since chemical treatment was not required. Strides have been made in the field of ECT over the decades. In fact, it is considered the most successful treatment for severe treatment‐resistant depression.^[^ ^17^ ^]^ Despite this, the preconception of ECT is generally poor,^[^ ^18^ ^]^ despite its efficacy and improved safety.^[^ ^16^ ^]^ Though this therapy has fallen into disuse, it is still considered in cases where the resources are limited, or clinicians are seeking to reduce the invasiveness of the treatment and avoid surgery.

An alternative type of ECT, the focal electrically administered seizure therapy (FEAST) was designed to achieve a better degree of focality and of efficiency of the electric pulse in order to mitigate the potential side‐effects of the therapy,^[^ ^19^ ^]^ such as headache, muscle pain, and memory loss.^[^ ^20^ ^]^ From initial animal studies,^[^ ^21^ ^]^ this technique was regarded as a promising anticonvulsant.

### 2.2 Transcranial Magnetic Stimulation

Transcranial magnetic stimulation (TMS; Figure 3B) is a powerful and painless procedure frequently used both in clinical and research practices to shed light on movement, vision, language, and neurological disorders.^[^ ^22^, ^23^, ^24^ ^]^

This technique involves a localized magnetic field focused on the brain capable of modulating cortical excitability (either increasing or decreasing it).^[^ ^22^ ^]^ Depending on the frequency, number of pulses, train duration, and intertrain intervals, the induced current activates a wide variety of neurophysiological and/or behavioral effects producing a benefit on the patient's brain.^[^ ^23^ ^]^

Nowadays, TMS seems to be relevant also for therapeutic practices in neurology, rehabilitation and psychiatric conditions, such as chronic depression and substance abuse disorders.^[^ ^25^, ^26^ ^]^

A modification to the previous technique is the repetitive transcranial magnetic stimulation (rTMS; Figure 3B) that involves prolonged and repeated magnetic stimulation (MS) of the brain through the scalp.^[^ ^27^ ^]^ During the rTMS treatment the magnetic coils switch polarity, with a period in the order of microseconds. This process creates a strong magnetic field, which in turn produces an increased response in the brain: this therapy is both efficient and fast.^[^ ^27^ ^]^ Depending on the frequency of the magnetic field applied, different areas of the brain will be stimulated with different intensities, and dictates whether the target region is inhibited or excited.^[^ ^28^, ^29^ ^]^ For example, using a 10 cm circular coil and targeting the cerebellum, it is possible to stimulate that region exclusively through proper antenna positioning. rTMS is used for anxiety and depression in people with treatment resistant depression,^[^ ^30^ ^]^ for postural control in people with spinocerebellar ataxia,^[^ ^31^ ^]^ and to reduce seizures.^[^ ^29^ ^]^

### 2.3 Micromagnetic Stimulation

Following the extensive interest surrounding optogenetics (see Section 2.5) in the research community, there has been investigation into the feasibility of magnetogenetics, in which genetically modified tissue would be receptive to magnetic stimulation.^[^ ^32^ ^]^ While this technique is still in its infancy, alternatives exist which have progressed beyond TMS: namely, μMS from implantable microcoils (Figure 3C). Numerous benefits arise from using μMS over the conventional electrode‐based stimulation. Firstly, the activity of selected neuronal subpopulations is better controlled due to the spatially asymmetric magnetic stimulation.^[^ ^33^, ^34^ ^]^ Second, the coil encapsulation with materials which have previously been designated as biocompatible, such as parylene^[^ ^35^ ^]^ and liquid crystal,^[^ ^36^, ^37^ ^]^ may reduce the neuroinflammatory response. Electrode performance degrades over time due to corrosion, and the increasing distance between the probe and its stimulation target, due to scarring and neuronal death. By contrast, magnetic fields are permeable to tissue and biological materials, and the coils will remain as effective stimulators in spite of the immune response and the scar formation.^[^ ^38^, ^39^ ^]^ Furthermore, avoiding the direct contact of the metal coil with the host tissue, this system overcomes several complications caused by the tissue‐electrode interface.^[^ ^40^, ^41^, ^42^, ^43^ ^]^

A fully encapsulated mm scale coil was tested in vitro, and confirmed to stimulate retinal ganglion cells.^[^ ^33^ ^]^ Miniaturization efforts yielded a coil measuring 500 µm in diameter × 1 mm long, which is comparable to the state of the art in electronic probes even for those implanted both in the cortex and deep subcortical nuclei. Successful stimulation with high selectivity was produced by altering the orientation and the magnitude of the coil relative to nearby neurons.^[^ ^38^ ^]^ The most attractive feature of implantable magnetic stimulation is the long‐term reliability and the fact that they are Magnetic Resonance Imaging (MRI) compatible due to a good electrical insulation of the coil that limits the heating induction.^[^ ^33^ ^]^ Concerns about the heat produced by the coils during operation have been addressed using finite element method (FEM) simulation which report that the temperature would not increase above 39 °C in the brain.^[^ ^39^ ^]^ However, following the example of DBS electrodes, the maximum allowable increase is 1 °C.^[^ ^44^ ^]^

### 2.4 Deep Brain Stimulation

The most extensively employed technique for neurostimulation is DBS (Figure 3D). It is an established treatment option that involves the placement into the brain of a medical device, also called “brain pacemaker,” for the improvement of motor problems associated with PD, essential tremor, and dystonia,^[^ ^45^ ^]^ as well as conditions including, but not limited to, epilepsy and clinical depression.^[^ ^46^, ^47^ ^]^ Pulses that mimic the natural flow of impulses are used to electrically stimulate neural pathways of specific areas of the brain (typically the motor cortex or cerebellum)^[^ ^46^ ^]^ providing motor function restoration, improvement in quality of life, and adequate control of movements in PD patients. However, this treatment is not suitable for every patient and thus, its success depends on the patient's condition.^[^ ^48^ ^]^ Electrodes may be inserted either side of the basal ganglia, or on one side only. Typical implantation sites are the subthalamic nucleus (STN‐subthalamic stimulation) or globus pallidus internus (GPi– pallidal stimulation), but the thalamus (thalamic stimulation) can also be a target location. Leads extended from the implanted pulse generator to the stimulating probe are often a cause of repeated surgeries, as they require position adjustment, treatment for skin erosion or infection, or replacement due to lead fracture.^[^ ^49^ ^]^ This is a potentially negative outcome of this implant typology. A typical DBS device includes a battery that supplies the controller and provides enough energy for the stimulation. As expected, there are many issues associated with this implant. Despite the necessity of MRI for many DBS patients, increased brain tissue temperature and current through the device represents a significant risk, as indicated by researchers’ efforts to review the hazards and collate best practice techniques to reduce patient harm.^[^ ^50^ ^]^ For example, finite element models may be used to evaluate the specific absorption rate (SAR) and heating experienced during MRI, without potentially endangering patients.^[^ ^51^ ^]^

The bioheat equation was first coined by Harry H. Pennes in 1948 and this equation models the changes in temperature of a human tissue considering parameters such as tissue density, tissue specific heat, temperature, time, tissue thermal conductivity, volumetric heat source (*Q*_v_), heat loss due to perfusion (*Q*_p_), and metabolic heat generation (*Q*_m_).^[^ ^52^ ^]^ The *Q*_v_ represents the effect of electromagnetic fields associated with transcutaneous energy transfer and transcutaneous telemetry. The *Q*_p_ term is the heat loss of the perfusion and how the cooling effect of the blood vessels affects to the temperature of the tissue. Finally, *Q*_m_ term is the metabolic heat generated by the tissue as an effect of the metabolic changes. For instance, brain tissue metabolic changes can vary from 0.5 to 4.0 nW depending on location and level of activity.^[^ ^52^ ^]^ In addition, the length of the implanted leads increases the likelihood of mechanical defects and immune response. The rigid, sizeable components used in a typical DBS system prevent successful biointegration.^[^ ^11^, ^12^, ^53^ ^]^ Although DBS in its current form is the most adopted technique, important advances are being made in order to address biocompatibility issues. The latest advances in wireless power transmission systems provide an opportunity to remove implantable batteries from the system entirely.

### 2.5 Optogenetics

Optogenetics is a photostimulation procedure which aims to achieve specific stimulation of the brain tissue, integrating optical and genetic detection techniques. It has been established as a promising alternative to traditional electrostimulation (Figure 3E). This novel method utilizes light‐sensitive proteins, such Channelrhodopsin‐2 (Ch2) which naturally occurs in *Chlamydomonas reinhardtii* alga.^[^ ^54^ ^]^ It is possible to genetically modify the specific neurons which will be the target of the stimulation with lentiviral gene delivery of this protein. Action potentials occur on the target cells in response to light stimulus characterized by a specific wavelength. The main advantage of this technique is its specificity.^[^ ^55^ ^]^ This is further explored in a review by Won et al., which discusses how electrostimulation cannot focus as effectively on cells or small brain regions.^[^ ^56^ ^]^ Different levels of tissue penetration are enabled by different wavelengths, while the target neurons may be stimulated or inhibited exclusively according to their genetic modification, with the absence of conducting wires on the brain tissue. Exposed stimulating electrodes may be the site of irreversible reactions as described further in Section 4. In their place, optogenetic systems utilize micro‐light emitting diodes (LEDs), which are often coupled with waveguides. However, micro‐LEDs have their own negative effects without careful duty cycle control, such as tissue heating.^[^ ^57^ ^]^ Replacing wires and probes with micro‐LEDs and optical waveguides removes the need for stimulating electrodes, which may be the site of irreversible reactions as described further in Section 4 Emerging technologies in the field of biochemistry will lead this new wave of stimulation techniques based on the protein‐function specificity.^[^ ^55^ ^]^ Using a wide range of light‐dependent‐proteins and optoelectronic devices, it is possible to stimulate specific neurons instead of whole areas of the brain.^[^ ^55^, ^58^, ^59^ ^]^ Particular emphasis must be placed on the light penetration into the brain tissue. Since there are many factors that determine the light penetration in a biological tissue, such as tissue density, light wavelength or power of the light source. Tedford et al.^[^ ^60^ ^]^ report a maximum light penetration of 45 mm from the light source into the brain tissue. The light source must be placed over the tissue as close as possible. In their report they used a 808 nm wavelength and 5 W laser.

In order to improve the penetration depth, near‐infrared (NIR) light has been used in optogenetic systems, moving away from the traditional LED‐based geometry. A detailed review was carried out by Yu et al., discussing how NIR light may be converted to visible light or heat inside deep brain regions, for the purposes of optogenetic or thermal stimulation.^[^ ^61^ ^]^ Light stimulation seems to be a very promising technique, removing the need for invasive probes and using a LED to stimulate the genetically modified neurons.

## 3 Bioinspired Design

### 3.1 Immune Response

In order to understand the best design practice for implantable neural probes, it is important to understand the mechanism of the foreign body reaction (FBR), which may require up to a month to stabilize after implantation,^[^ ^12^, ^62^, ^63^ ^]^ though in some cases the FBR has lasted a number of years.^[^ ^11^ ^]^ While the brain microenvironment is tightly regulated and protected by the blood‐brain barrier (BBB), neural probe implantation itself causes physical damage of host tissue (drilling into the skull, piercing the dura mater, and compromising the BBB and the extracellular matrix), which in turn induces an acute neuroinflammatory reaction, known as FBR.^[^ ^64^ ^]^ The FBR process involves the activation of the tissue‐specific innate defenses, microglia and astrocytes, and their transition from a resting into a reactive state, and drive peripheral immune cells (i.e., macrophages) to enter into the insulted area.^[^ ^12^, ^62^, ^63^, ^64^, ^65^ ^]^

**Figure 4:**
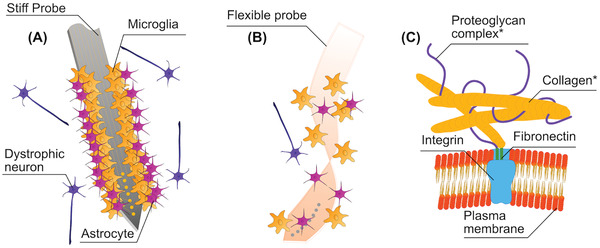
A) Immune response in the wake of rigid probe implantation. Astrocytes and Microglia adhere to the probe surface, as the foreign body response seeks to encapsulate the implant to protect tissues from further harm. Dystrophic neurons, which have been separated from neuron clusters and suffer degradation, will amass around the probe. B) Reduced foreign body response elicited by a flexible polymer probe. C) The plasma membrane on the outside of a cell is attached to *extracellular matrix (ECM) which is comprised of the proteoglycan complex and collagen fibers.

This process is illustrated in **Figure** **4**A. While microglia and macrophages locally deliver several proinflammatory molecules (e.g., cytokines, chemokines, interleukin‐1, nitric oxide, tumor necrosis factor),^[^ ^65^, ^66^ ^]^ astrocytes, connective tissue and extracellular matrix are primarily employed to the formation of a fibrous envelope, attempting to encapsulate the unfamiliar and potentially harmful device.^[^ ^13^ ^]^ Glial scars seek to defend the tissue surrounding a given implant displacing the neurons that are going to be recorded and/or stimulated;^[^ ^67^ ^]^ further, the scar is also interpenetrated with neurotoxic factors released from both microglia and astrocytes which hinder the regrowth and the recovery of target tissue.^[^ ^13^, ^41^ ^]^

The device/tissue crosstalk communication could also be affected in a long term phase due to the induction of the adaptive immune responses that could also be triggered by the implant deterioration (e.g., the change in the surface properties)^[^ ^68^ ^]^ compromising the device performance over time. This has major consequences for the noise of the recording, whereas for stimulation, a higher voltage will be required to excite the neurons.

Another important factor is related to the impact of aging that physiologically affects several features of the brain tissue, including the degeneration of neurons and oligodendrocytes,^[^ ^69^ ^]^ the viscoelastic properties of the cellular matrix,^[^ ^70^ ^]^ and the immunosenescence.^[^ ^71^ ^]^ These factors point some questions concerning the research of a proper design and biomaterials to satisfy different needs and diverse brain tissue response in terms of acute and chronic inflammatory reaction in aged versus young individuals.

The major challenge embraced by material science in developing neural interfaces is to understand the best design practice to improve the biological compliance with a view to minimize the initial trauma of healthy tissue and neuroinflammation in favor of the device‐host tissue interaction, and prolong the implant performance.^[^ ^72^ ^]^

### 3.2 Extracellular Matrix

The extracellular matrix (ECM) is a very dynamic meshwork of molecules, comprised of proteins and sugars (glycans),^[^ ^13^, ^73^ ^]^ released by neurons and glia in the interstitial space of tissues, as shown in Figure 4B. It has features that vary in size from 50 to 500 nm,^[^ ^74^, ^75^ ^]^ constituting 10–20% of brain volume.^[^ ^74^, ^75^, ^76^ ^]^ The ECM provides mechanical support to the structural organization of neuronal network and chemically sustains the normal physiology of the central nervous system (CNS), in terms of cell viability and neuronal activity.^[^ ^74^, ^75^ ^]^ It orchestrates several signaling processes throughout the development of the neural system and in the maintenance of synaptic plasticity in the adult mature brain. Pathological events that impair the normal ECM functions have been found to be linked with a wide range of neurological and psychiatric conditions, such as epilepsy^[^ ^77^ ^]^ and schizophrenia.^[^ ^78^ ^]^ Moreover, the ECM plays an active role during neuroinflammation processes.^[^ ^79^ ^]^

Considering that, tissue engineering employs ECM‐based materials for neural interfaces with the intent of simulate a substrate closer to the neuronal habitat in order to mitigate the immune response, reducing the scar formation and preventing cell death. The ECM coating includes a range of potential scaffold materials based on man‐made (e.g., Poly (ethylene glycol), PEG; Poly (glycolic acid), PGA; Polypyrroles (Ppys) and natural (i.e., collagen, fibrin, fibronectin, and hyaluronic acid) substances.^[^ ^80^ ^]^ The application of these materials depends on a host of physical and chemical characteristics. They have to be biocompatible and able to fit in the implantation site promoting the most favorable cell conditions. Moreover, these scaffolds should allow the optimal crosstalk communication with neural cells and ensure the long‐term device performance.^[^ ^74^, ^75^, ^80^, ^81^ ^]^

Neural implants which seek to mimic the composition of the ECM encourage neuronal penetration and regeneration, especially when employing polymers.^[^ ^12^ ^]^ This is of particular interest to tissue engineers, who focus on marrying biological scaffolds with cells for implantation in order to sustain the tissue integration and repair taking advantages of the molecules secreted from these cells which constitute the hybrid.^[^ ^73^ ^]^ Some of the most successful neural probes exploit a mesh‐like structure, with only 20% of the total surface area comprised of flexible substrate.^[^ ^82^ ^]^ Much like the support provided to neurons by the ECM, mesh probes encourage neuronal penetration.

### 3.3 Feature Size

Even if the implantation of an extraneous material always elicits a tissue reaction,^[^ ^83^ ^]^ there is ample evidence to suggest that limiting the dimensions of a neural probe to cellular or subcellular scale is the best practice to circumvent an immune response and provide a more accurate tissue integration and stimulation in a target area of the brain.^[^ ^84^ ^]^ Looking beyond the immune response, a smaller probe offers several other advantages in terms of tissue displacement impact, minimizing the steric blockage of signaling molecules and changes in the intracranial pressure due to the device volume in the host tissue and because of the penetration trauma and a possible complication caused by vasculature disruption, diminish the consequences of a cerebral edema that may be fatal.^[^ ^85^, ^86^, ^87^ ^]^ There is also concern over the performance of neurons following implantation: damage which extends over centimeters is likely to be permanent, as axonal recovery and regrowth has its limitations.^[^ ^67^ ^]^ While reducing the footprint of the device can have negative consequences for the impedance of the electrodes, coatings with organic conductive polymers such as poly(3,4‐ethylenedioxythiophene):poly(styrenesulfonate) with carbon nanotubes (PEDOT:PSS/CNT)^[^ ^88^ ^]^ may compensate for this, increasing the charge transfer capacity.^[^ ^89^ ^]^

In this regard, other materials such as iridium oxide,^[^ ^90^ ^]^ amorphous silicon carbide,^[^ ^91^ ^]^ and ruthenium oxide,^[^ ^92^ ^]^ which exhibit high charge injection capacity and low‐impedance coatings have been recently gained ample attention for the development of microelectrodes with both neural recording and stimulation performance.

### 3.4 Anchorage and Micromotion Dependence

As discussed in‐depth in a review by Discher et al., anchorage dependent cells require a solid substrate to adhere to: in vitro, both neurons and astrocytes are discerning when it comes to substrate stiffness, and their growth is affected accordingly.^[^ ^93^ ^]^ In fact, based on the differences in the biomechanical properties of neuronal and glial cells, while neurons in vitro grow well on soft, flexible surfaces, astrocytes fare better on rigid materials.^[^ ^94^ ^]^ Investigation into neuronal growth on poly(acrylic acid) (PAA) gel uncovered the fact that neurons successfully produce neurites, whereas glial growth was nonexistent.^[^ ^94^ ^]^ Methods of producing stretchable substrates include metal deposition onto a material surface while it is being stretched, or serpentine layouts,^[^ ^73^ ^]^ and as such there is opportunity to further investigate the effects of flexible substrates on neuronal growth in vivo.

Together with anchoring, the chronic stability of a neuromorphic device is influenced by micromotion. It is defined as small movements (i.e., submicron to micron) of the foreign tool in the native tissue related to several factors: mechanical interactions between the surrounding tissue and the electrode interface (mechanical sources),^[^ ^95^ ^]^ vibrations associated with entire body motion (behavioral sources)^[^ ^96^ ^]^ and movements caused by cardiac rhythm, breathing and vascular pulse (physiological sources).^[^ ^97^, ^98^ ^]^

Reinforcing the connection between the implanted device and the neural tissue, for example acting on the mechanical features of the materials, may contribute to the stability of the implant, decreases the microdamage and reduce the stress and strain at the electrode–brain interface as well as the FBR response at early stages.^[^ ^99^, ^100^, ^101^ ^]^ Finite element modelling has been used to suggest that an implant that moves with the brain tissue, for example in the case of neuronal interpenetration, will cause less damage due to micromotion: this requires significant validation in vivo before it may be treated as a design recommendation.^[^ ^102^ ^]^

As discussed in the design recommendations in Section 9.4., there is a case to be made that wired or tethered brain implants can have a significant impact, not only on the micromotion inside the brain,^[^ ^103^ ^]^ but the natural behavior of animals during experiment.^[^ ^58^ ^]^ Extensive research has been carried out by academic groups lead by Rogers,^[^ ^104^, ^105^, ^106^ ^]^ Sheng,^[^ ^107^ ^]^ and Degenaar,^[^ ^108^ ^]^ among others, on the development of wireless optogenetic probes. The breadth of work on the subject shows that the design effort required to remove wires is beneficial enough to incorporate into numerous systems. Working towards fully integrable neural implants, probes have been designed to “float,”, i.e., be tethered to connections outside the skull only by a thin and flexible polyimide cable.^[^ ^109^, ^110^ ^]^

### 3.5 Nanopatterning

The surface of the brain is not perfectly smooth: in an attempt to integrate successfully with neural tissue, implants will often be patterned with nano‐ or micronscale features. Different features of a neural probe surface, such as the topographical and roughness characteristics, may weaken the immune process,^[^ ^111^, ^112^, ^113^ ^]^ and improve chronic tissue integration. Detailed design strategies of the surface can provide a street map for in vitro nerve regeneration influencing the direction and extent of neuronal growth, which can be accomplished by molding polymers such as Poly(dimethyl siloxane) (PDMS) or polystyrene‐*block*‐poly(ethylene‐ran‐butylene)‐block‐polystyrene (SEBS).^[^ ^114^, ^115^ ^]^ For example, the depth of a channel dictates whether a neuron in vitro will grow along it, or orthogonal to it; axons grow more readily on raised areas compared to channels; and the radius of a fiber can shape the formation of neurites.^[^ ^116^ ^]^

### 3.6 Biological Coatings

Probe coatings have many uses, most commonly the modification of stiffness during implantation. However, they can also be biofunctional: as the coating degrades, drugs or even living cells will be exposed to the surrounding tissue, which can help to prevent inflammation, or aid integration.^[^ ^89^ ^]^ Typically, a hydrogel is employed to slowly and harmlessly degrade inside the body, and is attractive in terms of its noninterference with electronic recordings, and because a chosen layer thickness is easily reproducible. Hydrogels must be selected to degrade at an appropriate rate to prevent them from drawing in fluid from the surrounding tissue.^[^ ^117^, ^118^ ^]^ A “living scaffold”^[^ ^119^ ^]^ is described as a regenerative scaffold which combines living cells organized in 3D topology and biomaterials which can be used to replenish the cells in an area where tissue has been damaged,^[^ ^67^ ^]^ for example, areas of neuronal loss following implantation of a probe. The scaffold itself should be engineered to assimilate to the target tissue, encourage axonal growth, and provide structure for cell proliferation.^[^ ^119^ ^]^ There is some concern around the immune reaction to any foreign cells introduced into the scaffold; however, stem cells are reported to mitigate inflammation, and encourage tissue restoration.^[^ ^62^ ^]^ Glia in “proregenerative states”^[^ ^120^ ^]^ may also help to protect neurons from the detrimental effects of the FBR. The “living scaffold” is taken one step further with the advent of “living electrodes” or “micro tissue engineered neural networks”^[^ ^72^ ^]^ which shows significant progress towards artificial neurons which integrate harmoniously with real neurons.^[^ ^117^, ^118^ ^]^

Oxidative stress due to implanted microelectrodes represents another important aspect in neurodegeneration also influencing the device functioning.^[^ ^121^ ^]^ Use of antioxidative coating, such as small interfering RNA‐mediated gene silencing for specific genes,^[^ ^121^ ^]^ and superoxide dismutase (SOD) mimetic Mn(III)tetrakis (4‐benzoic acid) porphyrin (MnTBAP),^[^ ^122^ ^]^ may represent new therapeutic strategies to reduce the force of neuroinflammation and reactive oxygen species (ROS) production targeting on specific inflammatory pathways.

### 3.7 Hydrophobicity/Hydrophilicity Chemical Properties

The permeability of a medical device surface in terms of hydrophobicity and hydrophilicity is a critical material property that influences the energetic interactions between the outer face of the device and the protein, making the surfaces capable of interfering with the protein absorption.^[^ ^111^ ^]^

Biomaterial hydrophobicity could determine immunogenicity^[^ ^123^, ^124^ ^]^ through the recognition of the hydrophobic moiety of biological molecules by the immune system as a marker of a potentially harmful signal pathway.^[^ ^123^ ^]^ Hydrophobic substrates, in an aqueous biological medium, leads to disorder in the entropy of water molecules in favor of protein adsorption and aggregation to the biointerface. In this view, following the immune response, proinflammatory molecules, and neurotoxic cytokines are absorbed more extensively on the surface of such materials, and inflammation is increased over time perpetuating the chronic response.^[^ ^125^ ^]^ It has been suggested that hydrophilic coating may upgrade the biocompatibility by creating a water‐attracting surface, and diminishing the amount of absorbed protein compared to a hydrophobic interface.^[^ ^126^, ^127^, ^128^ ^]^ Moreover, the functional groups that are exposed on the material surface can differently affect the biological compliance of a device. The most represented are methyl (—CH_3_), amino (—NH_2_), hydroxyl (—OH), and carboxyl (—COOH) groups.^[^ ^129^ ^]^

There is some debate about the possibility that the surface charge may modulate the protein adsorption and their folding more than the physical properties of hydrophilicity and hydrophobicity, and as a consequence are integral to the immune response. In brief, the hydrophobic and neutral‐charged —CH_3_ moieties show to amplify and sustain an inflammatory response accompanied by increase in the scar thickness.^[^ ^130^ ^]^ Among the hydrophilic functional groups, —NH_2_ (which is positively charged) and —OH (neutral), elicit acute inflammatory response, cell infiltration and long‐term in vivo fibrotic reactions^[^ ^131^ ^]^ even if only —OH shows lower protein affinity while —NH_2_ reveals to enhance protein adsorption and denaturation.^[^ ^132^, ^133^ ^]^ On the contrary, the hydrophilic and negatively charged —COOH group may mitigate glial scarring and cell infiltration.^[^ ^131^ ^]^ These attributes are summarized in **Table** **1**.

**Table 1:** Summary of most common molecules on the surface of a biomedical implant, and the consequences of the presence of these groups

Group	Hydrophobicity	Charge	Action in the body	Reference
—CH_3_ (methyl)	Hydrophobic	Neutral	Inflammatory response, increased scar thickness	^[^ ^130^ ^]^
—NH_2_ (amino)	Hydrophilic	Positive	Inflammation, cell infiltration, fibrotic reactions	^[^ ^131^, ^132^ ^]^
—OH (hydroxyl)	Hydrophilic	Neutral	Inflammation, cell infiltration, fibrotic reactions	^[^ ^131^, ^133^ ^]^
—COOH (carboxyl)	Hydrophilic	Negative	Glial scarring and cell infiltration	^[^ ^131^ ^]^

In order to allow an implanted electrode to be effectively integrated in the host tissue, it is necessary to implement some strategies to reduce unsuitable tissue responses through proper surface modification techniques that remodel physical and chemical features of the implant outer surface.^[^ ^120^ ^]^

Since nonspecific protein adsorption has been suggested to give rise to an inflammatory response, for example Zwitterionic poly (sulfobetaine methacrylate) (PSB) and polydopamine (PDA) coated implants have been recently investigated as a promising material sharing a hydrophilic nature, to promote a reliable electrode‐tissue interface and suppress microglia activation and neuronal loss.^[^ ^134^ ^]^ Further, graphene‐based flexible microprobes may be used for the detection of neural and cardiac recordings. In particular, the hydrophilization treatment by introducing hydroxyl groups on graphene outer surface made the microelectrodes suitable for biological applications.^[^ ^135^ ^]^

Despite the fact that a modified, hydrophilic film may help to restrain the FBR,^[^ ^136^ ^]^ most of the common polymers employed in the fabrication of neural probes are, indeed hydrophobic (e.g., parylene‐C, SU‐8 and PDMS), but still largely biocompatible. As concluded by Lee et al.,^[^ ^63^ ^]^ the effect of hydrophobic groups on the surface of these polymers may, in reality, have an insignificant effect on the FBR. In the case of SU‐8, surface modification to increase the hydrophilicity aids in the delivery of mesh electronics in solution, delivered through a syringe.^[^ ^137^ ^]^

## 4 Probe Design

### 4.1 Recording and Stimulating Electrodes

When seeking to both record and stimulate a target area of the brain with a single electrical probe, it is crucial to understand the difference in design requirements for the two functionalities. Characteristics of a selection of recording, stimulating and bidirectional probes are summarized in **Table** **2**. Microelectrodes must achieve a high charge injection capacity (CIC), and are often physically larger and produced with different materials compared to recording electrodes, for example sputtered iridium oxide,^[^ ^138^ ^]^ or PEDOT:PSS coating.^[^ ^139^ ^]^

**Table 2:** Experimental and design characteristics of recording, stimulating, and bidirectional neural probe systems. The disparity in electrode size is of particular importance, with several examples of bidirectional probes with multiple different electrode diameters on the same shaft. CIC refers to charge injection capacity, while CSC is charge storage capacity

Probe type	Experiment type	Target	Experiment length	Overall implant size	Electrode size	Impedance	Materials	CIC	CSC	Histology	Reference
Stimulating	In vivo	Rodent dorsal hippocampus, 100 µm radius of activation	4 h of stimulation	16 microelectrode shafts × 33 µm diameter	N/A	N/A	Tungsten, polyimide insulation, platinum black electrodes	N/A	N/A	c‐fos, DAPI, NeuN	^[^ ^84^ ^]^
Stimulating	In vivo	Primary auditory cortex (A1)	1 week	Single shank, 16 site, 100 mm pitch NeuroNexus	N/A	200–1400 kΩ at 1 kHz	Silicon	N/A	N/A	Iba1	^[^ ^40^ ^]^
Bidirectional	In vitro	N/A	N/A	N/A	10–100 µm diameter	3.3–3.8 at 1 kHz	Polyimide, titanium, gold, iridium, or platinum electrodes	N/A	14–21.8 mC cm^−2^	Only basic cytotoxicity screening	^[^ ^142^ ^]^
Bidirectional	In vitro, in vivo	Medial septum, and along the septotemporal axis of the hippocampus	35 days	125 µm diameter, PTFE‐coated, PTT0502, World Precision Instruments	125 µm diameter electrode	66.71 ± 0.44 kΩ	Nanostructured platinum, polyimide	3.0 ± 0.1 mC cm^−2^	51.3 ± 0.2 nC	GFAP, with no comparison to uncoated wire	^[^ ^369^ ^]^
Bidirectional	In vitro, in vivo	Action potentials in the rat cortex	38 days	5 mm long, 130 µm wide at maximum, 30 µm thick	160– 4000 µm^2^	10 kΩ minimum at 1 kHz	Polyimide, PEDOT:PSS, silicon stiffener, gold microelectrodes	2 mC cm^−2^ in vivo, 1 mC cm^−2^ in vitro	N/A	N/A	^[^ ^110^ ^]^
Recording	Agar Phantom	N/A	N/A	1.3 mm long Si tip, 6 mm PI cable	15 or 25 µm diameter	1.44 MΩ at 1 kHz immersed in Ringer's solution	Polyimide, silicon, iridium oxide or platinum electrodes, gold wiring	N/A	N/A	N/A	^[^ ^109^ ^]^
Recording	In vivo	Neocortex	3 h	8 mm long, 100 µm wide, 50 µm thick	20 × 20 µm	50 kΩ	Silicon, titanium nitride	N/A	N/A	DiI, Nissl to verify probe position	^[^ ^346^ ^]^
Recording	N/A	Hippocampus, cortex	N/A	5.5 mm × 180 µm (hippocampus), 2 mm × 230 µm wide (cortex)	30 µm diameter	Below 10 MΩ at 1 kHz	Parylene‐C, platinum, titanium/aluminium	N/A	N/A	N/A	^[^ ^143^ ^]^

Barz et al.^[^ ^109^ ^]^ and Schander et al.^[^ ^110^ ^]^ both combine polyimide ribbon cables with silicon stiffened shafts. While Barz has recording electrodes limited to 25 µm diameter, the maximum stimulating electrode area used by Schander is 4000 µm^2^. As an overall size comparison, the stimulating silicon probe (130 µm wide, 30 µm thick) is less than double the width and of comparable thickness to the Neuropixels probe from Jun et al., which measures 70 µm by 20 µm.^[^ ^140^ ^]^ High electrode counts, such as those displayed by the neuropixels probe, provide the opportunity to record activity from multiple neurons at once. This may pave the way for greater understanding of how groups of neurons, and potentially even multiple brain regions, work in tandem.^[^ ^141^ ^]^

When considering polymers, Tooker et al. employ a polyimide substrate for their recording and stimulating probe, with multiple metal layers.^[^ ^142^ ^]^ Similarly, the impressively dense, parylene probe from Scholten et al. uses an electrode bilayer, also with platinum metallization.^[^ ^143^ ^]^ The bidirectional probe boasts electrodes with varying diameters between 10 and 100 µm, while the recording probe is limited to 30 µm. However, the comparison in Table 2 indicates that neither were fully evaluated in vitro.

The volume of tissue activated by a neural probe is correlated to its size,^[^ ^84^ ^]^ and the intensity of the stimulus.^[^ ^144^ ^]^ Once it has been confirmed that a probe will excite nearby neurons, it is necessary to limit the stimulation magnitude to prevent electrolysis in the biofluidic environment, tissue damage and degradation of the electrodes themselves.

Significant effort has been expended to ensure the safe operation of neural stimulus systems, such that researchers may prevent irreversible reactions at the electrode and harm nearby tissue. In order to modulate the charge injection from an electrode, current control is used commonly, but not exclusively.^[^ ^145^ ^]^ The significance of the Shannon criteria, which are used to set the maximum stimulation amplitude, cannot be overstated.^[^ ^146^ ^]^ This prolific work is used to define the parameters for countless neural probe experiments, and has been the basis for further investigation into tissue damage during microelectrode stimulation.^[^ ^43^ ^]^

### 4.2 Corrosion and Irreversible Reactions

As part of electrode characterization, accelerated electrical aging requires that a significant number of cycles (for example, 3.5 billion) be performed on the implantable device to investigate electrode corrosion.^[^ ^147^ ^]^ The maximum applied voltage which avoids electrolysis of water is 1 V,^[^ ^148^ ^]^ although cyclic voltammetry is often carried out at marginally lower voltages. A definitive study carried out in 1974 concluded that iridium and platinum are among the most robust electrode materials in terms of corrosion,^[^ ^149^ ^]^ and as such are preferred over gold. Despite this, gold is still commonly used either as a track metal,^[^ ^150^ ^]^ or as the electrode itself.^[^ ^151^, ^152^, ^153^ ^]^ In contrast, silver is considered to be toxic and is unacceptable as an electrode material.^[^ ^154^ ^]^

The interface between electrodes and brain tissue is through electrical stimulation, as covered before. However, neural communication in the brain occurs through ion transfer in an electrolytic tissue media, more specifically through neurotransmitters that activate and deactivate ion channels in the neurons cell membranes.^[^ ^155^, ^156^, ^157^ ^]^ The effect of the local field potential created by the electrodes produces dynamic changes in terms of ionic movements in the surrounding tissues. These movements trigger firing of excitable cells by the depolarization of the transmembrane voltage of the targeted neurons and ionic currents in the surrounding tissue. During stimulation, increased voltage can accelerate the process of corrosion of the materials (i.e., electrode degradation) or even produce changes in the conductivity of the surrounding neurons, a phenomenon intensively studied since it is one of the main failure issues of neural probes.^[^ ^11^, ^43^, ^53^ ^]^

Relatively high voltages can unbalance redox reactions on the tissue surrounding the electrode and accelerate the corrosion of the materials used. In addition, depending on the shape of the electrodes, other phenomena such as the appearance of bubbles around the electrode interface due to the electrolysis that can insert oxygen bubbles into the electrode interface or the appearance of double layers of shielding tissue that produces changes in the impedance of the surrounding tissue thus affecting negatively to the stimulation efficiency.^[^ ^11^, ^12^, ^158^ ^]^

### 4.3 Failure Modes

There are two avenues of failure for an implanted probe, which are inextricably linked. The first is a prolonged and vigorous immune response, which takes the form of glial scarring, the proliferation of astrocytes, microglia accumulation and inflammation.^[^ ^159^ ^]^ Eventually, neurons in the surrounding area will begin to die. Scar tissue will increase the impedance between the electrode and the targeted neuron, and the signal‐to‐noise ratio (SNR) will decrease.^[^ ^160^ ^]^ These responses are primarily caused by surgical trauma^[^ ^161^ ^]^ and micromotion after implantation.^[^ ^162^ ^]^ Achieving low impedance requires compromise in terms of increased probe surface area, in accordance with the resistivity equation. As such, the immune response is largely affected by the first two criteria: scale and shape.

The second failure method involves the mechanical or electrical failure of the probe itself, mainly through delamination or fracture, which is exacerbated by surface defects such as microcracks.^[^ ^163^ ^]^ Improved fabrication, not limited to techniques which ensure the reliable adhesion of substrate and conductor, reduces the risk of delamination. Thermal annealing is a useful method to accomplish this for parylene layers,^[^ ^164^ ^]^ while surface roughening,^[^ ^110^, ^165^ ^]^ or liquid adhesion promoter may be used for polyimides,^[^ ^166^ ^]^ depending on the second material the polyimide layer is attached to. Encapsulating the probe serves to increase the buckling strength compared to uncoated probes.

**Figure 5:**
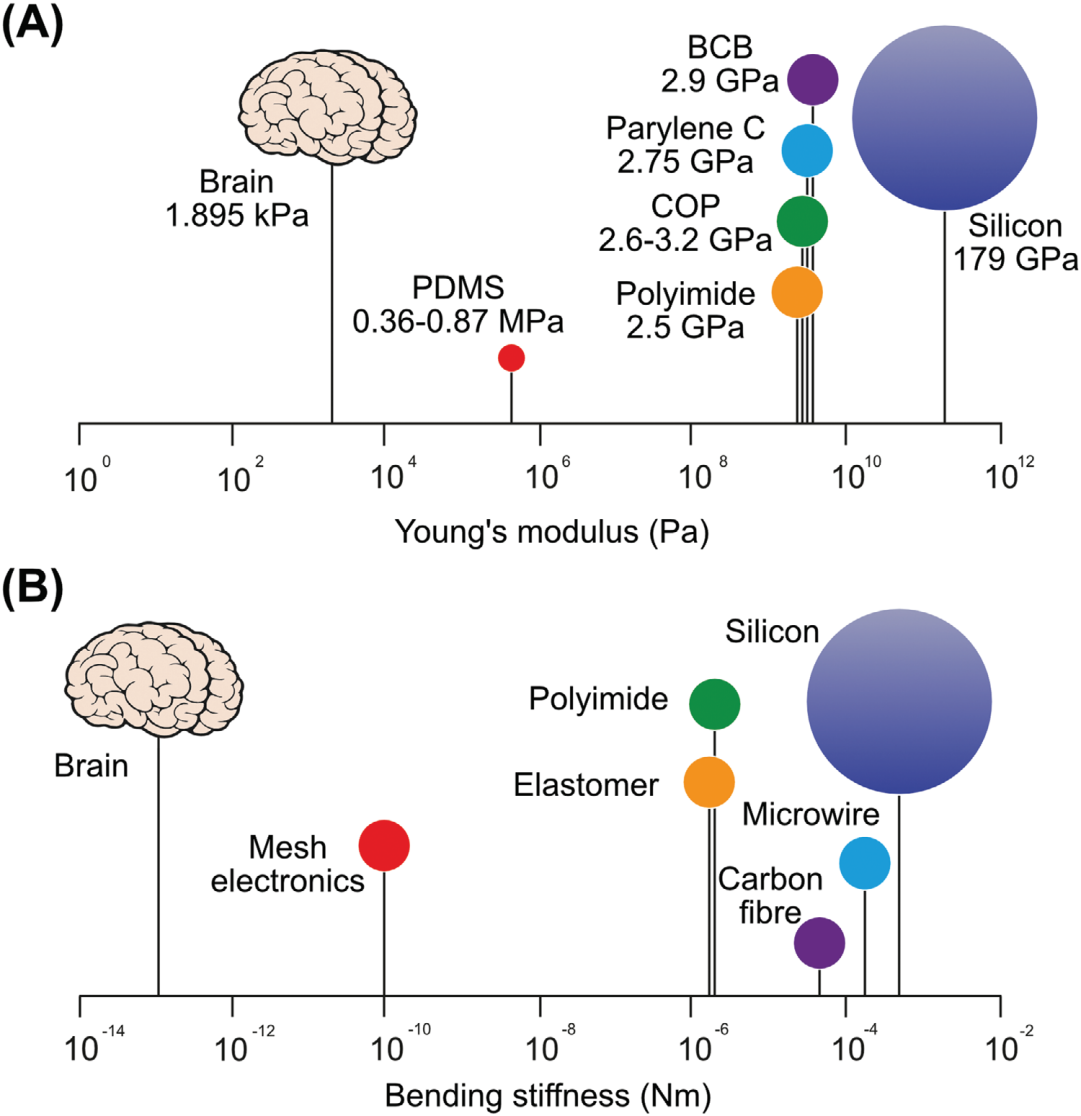
A) Young's modulus on log scale for brain tissue and neural probe materials, with silicon as the stiffest common substrate. B) The effective bending stiffness values of the most relevant implantable probe styles.

However, careful consideration must be made before selecting the encapsulation material. Without sufficient stiffness, the implant cannot be inserted into the brain; if the Young's Modulus of the probe is much greater than that of brain tissue (in rodent and human brains the *E* value has been identified in the range of 0.1–16 kPa),^[^ ^167^, ^168^, ^169^, ^170^ ^]^ trauma is more likely to occur. In **Figure** **5**, the Young's modulus of implant topographies is compared, alongside the bending stiffness of materials employed historically in neural probes. Further to this, the optical characteristics must be explored,^[^ ^171^ ^]^ while novel prospects such as drug delivery or medicinal properties also bear consideration.^[^ ^172^ ^]^

### 4.4 Miniaturization and Material Selection

Miniaturization of the implantable neural device allows the electronics to conform more closely to the surrounding tissue, and results in greater compliance, at the cost of considerable reduction of the buckling force. Reducing the thickness of stiff, e. g. silicon‐based probes, with high Young's modulus, reduces the disparity between the probe and neural tissues. The size of the probes made from these materials can be reduced to make microwires; for single microwire only one recording site can be made per shaft,^[^ ^173^ ^]^ but for the silicon‐based microprobe, thanks to the novel microfabrication techniques, up to 960 recording sites can be integrated on a 20 µm thick and 70 µm wide probe.^[^ ^140^ ^]^ Exploiting boron etch‐stop techniques, it is possible to fabricate a silicon probe shank with the thickness ranging from 5 to 20 µm.^[^ ^174^, ^175^ ^]^

In order to prevent mitigate blood vessel damage,^[^ ^176^ ^]^ as well as an immune response and neuronal death,^[^ ^177^ ^]^ the size of the shank and its delivery aid should be reduced as much as possible.^[^ ^178^, ^179^ ^]^ The surgical trauma induced during implantation should be encouraged to heal, not only through appropriate surgical techniques, but the minimization of the incision dictating the scale of the probe itself. Miniaturized features can also increase the number of electrodes per shaft.^[^ ^180^ ^]^ Importantly, there is some evidence that miniaturized probes may still prevent an immune response regardless of the rigidity of the chosen material at cellular scale.^[^ ^181^ ^]^ Neural tissue is very soft with respect to the materials used for neural probes. For example, the bending stiffness for a 20–100 µm thick brain slice is estimated to be 10^−13^ to 10^−10^ N m per unit width^[^ ^177^ ^]^ while for a neural probe made with silicon, this value rises to 10^−4^ N m. Figure 5 shows the different bending stiffness for different types of the neural probes. Using several layers of electrodes is shown to maximize flexibility in the case of two nanoelectronic thread probes with cross sections of 1 × 50 µm (inspired by silicon probes) and 1.5 × 10 µm (based on tetrodes) respectively.^[^ ^182^ ^]^ Commercial silicon‐based probes (e.g., NeuroNexus) with 15 or 50 µm thickness are available. It should be noted the thin brittle shanks of these probes are prone to fracture.^[^ ^178^, ^179^ ^]^

The length of the probe shank depends on the target location in the brain, as well as the species in which the probe will be implanted. For example, the probes which are implanted into the hippocampus of rodents are much smaller than those which have been used for human brain stimulation.^[^ ^183^, ^184^ ^]^ The main factor affecting the probe width is the number of required recording sites, which consist of the connections wires and pads.^[^ ^175^ ^]^ Therefore, miniaturization of the neural implant is restricted by the subject species, the number of the recording sites and the targeted point of the brain. It is also important to note that as the length of the probe increases relative to its width, it becomes less robust, increasing complexity and difficulty during implantation.^[^ ^185^ ^]^

**Figure 6:**
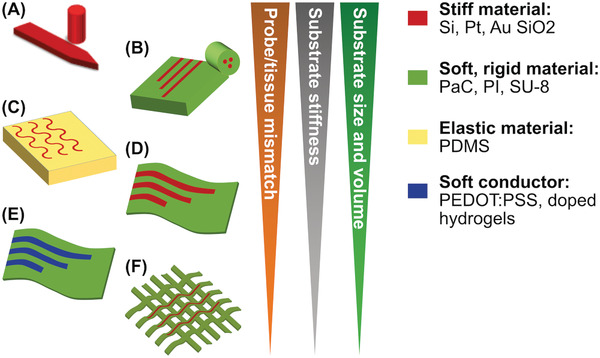
Evolutionary trend of the neural probe shape and material from very stiff needle‐like shaft to soft material‐based mesh/neuron‐like structures. A) Stiff shank or needle‐like neural probe, B) neural probe with thick fiber and polymer substrate, C) stretchable probe with serpentine metallic structures, D) ultrathin substrate‐based probe, E) ultra‐thin polymer‐based probe with soft conductive material, and F) mesh‐based probe.

Different neural probes with various forms and materials have been developed and demonstrated so far for neural activity recording, stimulation and manipulation purposes. An overview of these technologies is provided in **Figure** **6**. To decrease the immune response and mitigate the negative effects of micromotion in the brain, a shift away from stiff and needle‐like probes is crucial. The neural device can be made of soft material (e.g., polymeric or elastomeric), which provides lower mechanical mismatch with the surrounding neural tissue than stiff shanks (see Figure 6B). In some applications, a stretchable neural device is necessary. Elastic materials, serpentine structures, and mesh‐like neural devices can all be used to achieve this (Figure 6C,E). In this section, we review the evolutionary trend of the neural probes in terms of shape and material: from a very stiff needle‐like shaft to soft/stretchable materials and mesh/neuron‐like structures. It should also be noted that despite this remarkable structural transformation and its gains, rigid shanks and needle‐like probes such as Michigan or Utah array are still being used mainly because of their technological maturity.

### 4.5 State‐of‐the‐Art Probe Designs

#### 4.5.1 Stiff Probes

A platinum tungsten based tetrode with four probes (diameter of the metal measuring ≈30 µm) has been widely used to record neural signals. Electrical potential from 1100 neurons can be recorded using this probe in the neural tissue.^[^ ^186^ ^]^ Neural activity recording using a tetrode in a neurorobotic system, for various purposes such as identifying movement‐related information (e.g., in insect and rat), has been successfully demonstrated on a few occasions.^[^ ^187^, ^188^ ^]^ The Utah array is widely used in neural interfaces, and consists of several probes made of stiff material, e.g., silicon (see Figure 6A), which record only at the tip of the shank rather than employing electrodes along the entire length. Successful applications of the Utah array include neuroprosthetic arm control, and motor cortex recording to facilitate stimulation of the spinal cord in monkeys,^[^ ^189^, ^190^ ^]^ as well as clinical demonstrations for the rehabilitation of tetraplegia.^[^ ^191^ ^]^ Restoring hand movements, bypassing the spinal cord circuit, robot arms and neuromuscular controlling, are all further examples of the Utah neural probe as used in brain machine interfaces. The main drawback of Utah probes and tetrodes is their unsuitability for deep neural target recording. Michigan probes consisting of several neural electrodes, and typically with multiple recording sites along the shank, are versatile devices to be utilized in brain machine interfaces for different applications.^[^ ^192^ ^]^ Compared to the Utah probe, the Michigan probe (with a 3–15 mm long shaft) is better suited to activity recording in deep neural tissues. For instance, treatments based on stimulation of the neurons in a rat brain,^[^ ^193^ ^]^ and restoring missing brain function have already been demonstrated.^[^ ^194^ ^]^ The wealth of experience surrounding Michigan probe fabrication also creates an opportunity to include other functionalities on the shaft, e.g., LEDs to create an optoelectrode.^[^ ^195^ ^]^

#### 4.5.2 Polymer‐Based Probes

Fibers have shown good potential for use in neural devices, e.g., optical fibers to conduct light into a neural tissue from an outside source (see Figure 6B). A fiber probe has been developed to simultaneously perform neural signal recording, optical stimulation and delivery of drugs in freely moving mice.^[^ ^196^ ^]^ Utilizing this probe, which has been made from polymer using a thermal drawing technique, the authors have carried out neural signal recording and stimulation for two months. Compared to the shank or needle‐like array probes, polymer‐based probes lead to reduced FBR.^[^ ^196^ ^]^ Implantable polymer neural probes (Figure 6B) have also been demonstrated for neural activity recording, stimulation (such as a waveguide made with SU‐8 positive photoresist) and (polyimide‐based) fluid delivery in mice.^[^ ^197^ ^]^ In this work,^[^ ^197^ ^]^ a multimodal neural interface prototype enables transfer of electrical signals, fluid, and light for long term applications.

#### 4.5.3 Elastomeric Probes

For some applications (e.g., neural signal recording in the spinal cord) the probe must withstand local stretching. A soft and stretchable neural probe has been introduced for chronic neural signal recording in the CNS.^[^ ^198^ ^]^ Since the probe has been made with elastic material, in addition to required static properties, it meets the demands for dynamic mechanical properties. PDMS encapsulated optogenetic probes can be used to control neuronal activities using targeted proteins (see Figure 6C).^[^ ^199^ ^]^ The authors concluded that this biocompatible, soft and highly stretchable device (e.g., 10 times more stretchable compared to similar probes) enables experiment in various neural tissue in the body (i.e., peripheral and spinal pain circuits) and for long term applications.^[^ ^93^ ^]^

While a single PDMS encapsulation layer is not sufficient to prevent moisture ingress, there are many strategies which can be used to improve its suitability: roller casting instead of spin coating to prevent defects; multiple thin layers; combining PDMS with another polymer layer such as parylene to address the problem of water vapor permeability.^[^ ^200^ ^]^ In the case of Shelly et al. the PDMS implant was projected to last five years in vivo, but only after incorporating a titanium case to protect the electronics inside.^[^ ^200^, ^201^ ^]^

#### 4.5.4 Ultrathin 2D Neural Devices

Ultrathin planes of rigid polymers (e.g., polyimide, parylene, and polyethylene terephthalate) can be manufactured with the thicknesses ranging from 1 to 10 µm (see Figure 6D). Despite their higher elastic moduli (GPa) with respect to the elastomeric material (e.g., PDMS with modulus in the order of MPa), their ultralow thickness enables a very low bending stiffness. These 2D neural devices can be applied in cases where the device lies on the surface of the neural tissues, for example in subdural electrocorticography. But it should be noted, there are some 2D penetrating neural probe applications which have also been demonstrated.^[^ ^202^, ^203^ ^]^ Electrophysiological signals have been recorded on the surface of rat brain using an ultrathin parylene‐based 2D probe with the thickness of 4 µm,^[^ ^204^, ^205^ ^]^ demonstrating that ultrathin flexible and highly biocompatible organic transistor can record low‐amplitude brain activity.

Liquid crystal elastomers (LCEs) are a class of smart materials that reversibly change shape when exposed to a variety of stimuli, such as heat, light, or solvent. LCEs, as a subclass of liquid crystal polymers,^[^ ^206^, ^207^ ^]^ are shape‐changing polymers that possess liquid crystalline order and rubber elasticity. Their low elastic modulus and large shape change, up to 300–400%,^[^ ^207^ ^]^ have made them promising candidates to be used as substrate for implantable neural devices.^[^ ^208^ ^]^ The shape change of some LCEs can be programmed and this feature helps to develop 2D electronic during the processing and then create a programmed 3D shape.^[^ ^209^ ^]^ This feature of LCEs enables the controlled deployment of small recording sites to regions beyond that of the foreign body response induced tissue encapsulation zone surrounding an implanted shank.^[^ ^182^ ^]^ LCE's encouraging history of biocompatibility, high reliability under harsh working condition, and low moisture permeability, indicates that they are viable option for implantable neural devices.^[^ ^37^, ^210^, ^211^ ^]^

#### 4.5.5 Mesh‐Based Neural Devices

Mesh‐based probes are some of the most unconventional designs to have emerged in recent years (Figure 6F). Benefitting from their extremely low volume and flexible mesh‐like structure, these probes can perfectly blend to the surrounding neural tissues. One example of direct electrical recording of neural action potential in rodent brains utilizes subcellular feature sizes to overcome the and mechanical mismatches and limitations for long term applications.^[^ ^82^ ^]^ The probe consists of metal interconnects and recording sites including Pt electrodes and transistors sandwiched between SU‐8 layers with a thickness of 1 µm. The neuron‐like electrode (NeuE) mimics the structural features of the neuron and has been fabricated and applied for electrophysiology.^[^ ^179^ ^]^ The NeuE structure comprises metal sandwiched between layers of polymer with the total thickness of 0.9 µm. The smallest device polymer and metal widths are 1 and 0.6 µm, respectively.

#### 4.5.6 Mechanically Adaptive Probes

Temporary stiffening of the probe shaft during insertion is almost essential for flexible probes with a long, thin shaft, and in the case of those made from mechanically adaptive materials, the goal is to produce a probe which does not require any external insertion aid to achieve this rigidity. Two key approaches recruit the warm, fluidic environment inside the body to alter the composition of the probe: shape memory polymers (SMPs), with glass transition temperatures that depend on whether the material is wet or dry;^[^ ^212^ ^]^ and nanocomposite polymer matrices, in which the strong bonds between nanocrystals are broken in the presence of water.^[^ ^213^ ^]^

The Voit group have worked extensively with SMPs, emphasizing thiol‐ene in their body of work,^[^ ^214^, ^215^, ^216^, ^217^, ^218^, ^219^, ^220^, ^221^ ^]^ with a specific focus on the best fabrication practices to prevent delamination and thin film stress. In one notable example,^[^ ^222^ ^]^ combining a gold adhesion layer and iridium electrodes with a parylene layer to prevent moisture ingress, the in vitro study comparing a thiol‐ene‐based probe to a fully parylene probe showed significant reduction in the Young's modulus (over a factor of 10), and the presence of immunoglobulin at the implant site.

Nanocomposites of cellulose rods set in a soft, typically biocompatible polymer such as poly(vinyl alcohol)^[^ ^223^ ^]^ (which has been shown in vivo to produce only mild irritant effects)^[^ ^224^ ^]^ have been employed as polymer substrates for intracortical probes. In the first instance, Jorfi et al. indicate that during chronic implantation in vivo, the mechanically adaptive composite notably reduces the foreign body response over a time scale of 16 weeks when compared to a stiff probe.^[^ ^223^ ^]^ The Young's modulus reduces by a factor of 40 when soaked in water. Further to this, a functioning neural probe produced by Hess‐Dunning and Tyler^[^ ^213^ ^]^ with an even further decreased Young's modulus of 10 MPa was used to record single unit activity (SUA) in vivo for 16 weeks.

## 5 Encapsulation

The characteristics of polymer substrates and encapsulants are summarized in **Table** **3**, which highlights the most important selection criteria. For an in‐depth review of materials, with a view to designing neural probes for permanent implantation, the reader is also directed to the reviews by Song et al.^[^ ^225^ ^]^ and Jeong et al.^[^ ^226^ ^]^ Many researchers have identified polymer probes as the future of neural implants; however, special attention must be paid to biocompatibility testing of each new material, and application.^[^ ^227^ ^]^

**Table 3:** Material properties of the most widely adopted flexible substrate and encapsulation materials including: Young's modulus, target tissue, fabrication approaches, layer thickness, and transparency. Polyimide often boasts excellent biocompatibility, coupled with simple fabrication requirements and the lowest single layer thickness. PDMS has a greatly reduced Young's modulus compared to the other materials

Material	Young's modulus [GPa]	Target tissue	Fabrication	Layer thickness [µm]	Transparency	Hydrophobicity
Parylene‐C	2.75^[^ ^370^ ^]^	Rat motor cortex,^[^ ^229^ ^]^ mouse visual cortex^[^ ^4^ ^]^	Vapor deposition^[^ ^371^ ^]^	0.04–83^[^ ^372^ ^]^	94.7% optical transmittance^[^ ^230^ ^]^	Super hydrophobic^[^ ^262^ ^]^
PDMS	3 × 10^−4^ − 1 × 10^−3[^ ^373^ ^]^	Mouse hippocampus,^[^ ^234^ ^]^ rat sciatic nerve^[^ ^374^ ^]^	Vacuum plasma system^[^ ^179^, ^375^ ^]^	40^[^ ^179^ ^]^	Optically clear^[^ ^376^ ^]^	High^[^ ^376^ ^]^
Polyimide	2.5^[^ ^89^ ^]^	Rat cortex,^[^ ^110^ ^]^ mouse brain surface^[^ ^256^ ^]^	Spin coating and curing^[^ ^377^ ^]^	1–5^[^ ^378^ ^]^	Obviously transparent, transmitted above 370 nm^[^ ^379^ ^]^	82°^[^ ^380^ ^]^ therefore not hydrophobic since *θ* < 90°^[^ ^381^ ^]^
BCB	1.9^[^ ^382^ ^]^	Rat cortex^[^ ^383^ ^]^	Spin coating, followed by reactive ion etch or E‐beam^[^ ^248^ ^]^	20^[^ ^248^ ^]^	Transmittance of 70–80% in optical range^[^ ^384^ ^]^	Yes^[^ ^162^ ^]^
COP	2.6–3.2^[^ ^385^ ^]^	Rat somatosensory cortex^[^ ^151^ ^]^	CO_2_ laser^[^ ^151^ ^]^	13–188^[^ ^151^ ^]^ 7^[^ ^386^ ^]^	91%^[^ ^385^ ^]^ High between 300 and 1200 nm^[^ ^386^ ^]^	88°^[^ ^387^ ^]^
SU‐8	3^[^ ^388^ ^]^	Mouse somatosensory cortex^[^ ^182^ ^]^	Spin coating and curing^[^ ^159^ ^]^	0.9^[^ ^159^ ^]^–2^[^ ^256^ ^]^ µm	Optically transparent above 400 nm^[^ ^389^ ^]^	Hydrophobic^[^ ^390^ ^]^

### 5.1 Parylene‐C

Parylene‐C is a very common encapsulating material, which is described as flexible (Young's Modulus 2.76 GPa), and for many applications, shows encouraging results in biocompatibility tests (“ISO 10993 USP Class VI biomaterial”).^[^ ^12^ ^]^ It is an attractive encapsulant due to its conformability and biostability,^[^ ^228^ ^]^ which facilitates successful in vivo neural recording for a reported maximum of 12 months when used as both a substrate and an encapsulant.^[^ ^229^ ^]^ It is combined with an encapsulating SU‐8 layer by Seo et al. for acute in vivo recording.^[^ ^4^ ^]^ Parylene‐C is also transparent which is useful in that the tissue can be easily seen through the encapsulation material.^[^ ^4^ ^]^ Alternative applications for parylene also include the fabrication of transparent electrodes such as those made using conductive nanowires:^[^ ^230^ ^]^ parylene‐C offers both flexibility and stability, even in biofluids.^[^ ^231^ ^]^ Despite this, it exhibits poor adhesion (either between parylene‐C layers, or parylene‐metal) which makes parylene‐film devices vulnerable to moisture ingress during implantation, or delamination during fabrication due to extended immersion in solvents.^[^ ^232^ ^]^ Low temperature processing is required to prevent thermal stress in the polymer, which could cause the final device to be curved.^[^ ^143^, ^233^ ^]^ While most traditional nanofabrication techniques are unsuitable for parylene‐C processing, reactive ion etching is readily employed, while improved surface adhesion may be achieved through plasma enhancement.^[^ ^232^, ^233^ ^]^

### 5.2 Polydimethylsiloxane (PDMS)

PDMS is often used to create a microfluidic channel (e.g., for drug delivery),^[^ ^234^ ^]^ since it has a high viscoelasticity. PDMS has been shown to be safe for many existing implantable applications, the best‐known of which includes breast augmentation. Silicone has undergone significant scrutiny in the decades since its FDA approval,^[^ ^235^ ^]^ though biocompatibility testing is a crucial stage in the development of any new neural implant. For example, the curing agent which is used to solidify PDMS may be toxic.^[^ ^236^ ^]^ The immune response evoked by a PDMS implant may be mitigated using a PAA coating, which through careful surface design prevents cells from attaching to the implant.^[^ ^237^ ^]^ By increasing the device attraction to water, a hydrophilic coating reduced the immune response after implantation by regulating protein absorption. During in vivo tests, the PAA coating curtailed inflammation, with cells unable to cling to the implant. While the low Young's modulus of PDMS presents a challenge in terms of implantation, this remains a positive feature of the material. Bearing in mind the fact that tissue may recover from the trauma of a stiff implant if it is promptly removed, a PDMS substrate would be best paired with a stiff shuttle. Without any requirement on material rigidity, this presents new opportunities in terms of utilizing ultrathin, flexible ribbon cables, and opening the structure of the implant, perhaps with a macroporous design. PDMS is simple to prepare in the lab, and the biocompatibility of the material may be modulated by changing the ratio of PDMS elastomer to curing agent or boiling cured PDMS in water prior to insertion.^[^ ^238^ ^]^

### 5.3 Polyimide

The polyimide group encompasses a range of polymers which, for the purposes of neural probe fabrication, may be grouped into photodefinable (suffering from increased water uptake, as described by Stieglitz)^[^ ^239^ ^]^ and nonphotodefinable, as a preliminary classification. While Kapton is one of the most well‐known and widely applicable polyimides, neural probes are commonly manufactured using PI 2611 (also known as BPDA/PPD and U‐Varnish‐S). Despite the fact that polyimides are not FDA (Food and Drug Administration) approved,^[^ ^12^ ^]^ they have been evaluated by researchers at the Lawrence Livermore National Lab using ISO 10993 standards,^[^ ^142^ ^]^ as well as being USP Class VI compliant. Testing in water, phosphate buffered saline (PBS),^[^ ^240^ ^]^ in vitro assays^[^ ^239^, ^241^ ^]^ and in vivo studies extending up to 32 weeks,^[^ ^242^ ^]^ suggest that polyimides are largely biocompatible for long‐term applications. In order to improve cell adhesion, careful curing at high temperatures (above 300 °C) in an inert atmosphere evaporates all remaining solvent from the film surface.^[^ ^241^ ^]^ Polyimides are a strong choice for an encapsulation material, not least in terms of their low single layer thickness, which necessitates multiple rounds of spin coating to achieve a meaningful thickness above a few micrometers.

They are a popular choice for flexible ribbon cables, employing: Durimide 7510 for implantation of the sciatic nerve of a rabbit;^[^ ^243^ ^]^ PI 2611 which was submerged in PBS at body temperature for one month and evaluated in vitro;^[^ ^244^ ^]^ U‐Varnish‐S implanted into the cortex of Macaque monkeys;^[^ ^245^ ^]^ and U‐Varnish‐S for insertion tests into agar gel.^[^ ^109^ ^]^ Polyimides are readily implantable as a substrate material and would be more likely to withstand insertion force than PDMS. While polyimides are generally flexible but not stretchable, a novel patterning method which introduces open slits in the Durimide 7505 film allows the probe shaft to be stretched by up to 11%.^[^ ^246^ ^]^ Moving away from traditional planar probe shapes, a polyimide has also been utilized by Soscia et al. to produce a 3D array of recording probes, evaluated for more than one month in vitro, which provides a flexible alternative to a Utah array.^[^ ^247^ ^]^

### 5.4 Benzocyclobutene

Benzocyclobutene (BCB) is an attractive choice for a flexible substrate. With low water uptake, customarily high biocompatibility, and reduced layer thickness, dry‐etch BCB is an attractive substrate material. Biological materials may be incorporated on the probe surface, owing to the properties of BCB. In a notable example,^[^ ^248^ ^]^ the focus lies on the fabrication of a BCB neural probe, followed by evaluation of the thin film. In this case, high layer uniformity was achieved through rigorous improvement of the deposition and spinning process. The final thickness of the probe is quoted as 40 µm. BCB is classified as a substrate in its infancy, which requires further experiment.^[^ ^12^ ^]^

### 5.5 Cyclic Olefin Polymer

Cyclic Olefin Polymer (COP) has been employed for microfluidic applications, offering a reliable seal when the dimensions of the microchannel features are carefully controlled,^[^ ^249^ ^]^ and exhibiting negligible water permeability.^[^ ^250^ ^]^ An important consideration for COP films is the buckling strength, as highlighted through experimentation.^[^ ^151^ ^]^ For a COP probe with a width of 650 µm, much wider than a typical polymer shaft, the yield stress increases from ≈8 mN for the 50 µm thickness case, to around 220 mN for the 200 µm thick film. Although the neural recording capability was satisfactory, no comment was made on the longevity of the implant fabricated by Shim et al.^[^ ^151^ ^]^ Cyclic olefin copolymers (COC) are a variation on COP, in which the final polymer is produced from multiple monomers, such that the material properties may be tuned to include, for example, an “antioxidant and lubricant,” as produced by Bernard et al. and evaluated for in vitro cell toxicity.^[^ ^251^ ^]^ Notable recent examples of COC neural probes include: the work of Baek et al., comparing two films with different glass transition temperatures patterned with an aluminium mold;^[^ ^252^ ^]^ and a multimodal probe (incorporating chemical, electrical and optical sensing) using a COC to form a microfluidic channel.^[^ ^196^ ^]^

### 5.6 Chitosan

With origins in nature, as an increasingly common biomedical material, chitosan is a pragmatic choice for encapsulation of a neural probe. It is versatile, employed in hydrogels,^[^ ^253^ ^]^ medical dressings to help prevent infection,^[^ ^254^ ^]^ and cancer drug delivery,^[^ ^172^ ^]^ to name a few applications. Chitosan is biodissolvable but can be mixed with (3‐glycidyloxypropyl) trimethoxysilane (GOPS) to create a nondissolvable coating, or poly(vinyl alcohol) (PVA) to produce a very strong film. The main focus of some encouraging investigation was to successfully show that a chitosan outer coating would not affect neural recordings and would aid in identifying the probe during histology.^[^ ^255^ ^]^

### 5.7 SU‐8

Many of the neural implants which recruit the negative photoresist, SU‐8, exploit its flexibility and excellent biocompatibility for many existing applications. Low cost, easy patterning process and chemical stability are also important factors to consider. As part of the iWEBS probe, an SU‐8 encapsulation layer was used to facilitate smooth insertion of the multielectrode array (MEA) into a slit in the dura, and promote conformability on the brain surface.^[^ ^256^ ^]^

Standard photolithography is an obvious choice for SU‐8 processing. However, additional fabrication steps may be used to realize surface patterning, such as nanospheric lithography, or to create microfluidic channels, such as thermocompression.^[^ ^257^ ^]^ Microchannels of SU‐8 are also implemented in mm‐scale implantable scaffolds which encourage nerve repair.^[^ ^258^ ^]^ In both cases, cell interaction with the implant surface is imperative to its functionality. The roughened, biocompatible surface produced by Kim et al. showcases significantly increased neuron‐like cell adhesion and growth when compared to an unpatterned SU‐8 surface.^[^ ^259^ ^]^

Márton et al. performed a definitive in vivo study on SU‐8 only probes (with no electrode metallization) lasting 8 weeks.^[^ ^260^ ^]^ The most significant result lies in the radius of the neuronal “kill‐zone,” which for comparative silicon probes reached between 50 and 200 µm. Histological staining around the SU‐8 probe revealed no discernible neuronal death only 40 µm from the implantation site, with a glial scar only 5–10 µm thick. Despite significant astrogliosis in deep brain layers, the overall conclusion of their work was that SU‐8 is sufficiently biocompatible for their application.

One notable example takes advantage of the submicron layer thicknesses of SU‐8 to create a wrappable microelectrode array, which bends around a larger probe to create a nanoelectronic coating (NEC).^[^ ^261^ ^]^ Despite the generous size of the gold and platinum electrodes (30 µm × 30 µm), in vivo evaluation of the NEC facilitated recording of extracellular action potentials from the somatosensory cortex of mice.

Possibly the most successful application of an SU‐8 substrate, mesh electronics from the Lieber group have been proven to elicit no discernible foreign body response even after 12 weeks of implantation.^[^ ^159^ ^]^ Combining layers of SU‐8 (which total 900 nm thickness) with gold and platinum metallization on 20 µm wide polymer “lines,” mesh electronics are thin, conformable, and incredibly flexible. The longitudinal stiffness is equal to only 0.104 nN m, compared to 3300 nN m for a 500 µm wide polyimide film sample.

## 6 Fabrication

**Figure 7:**
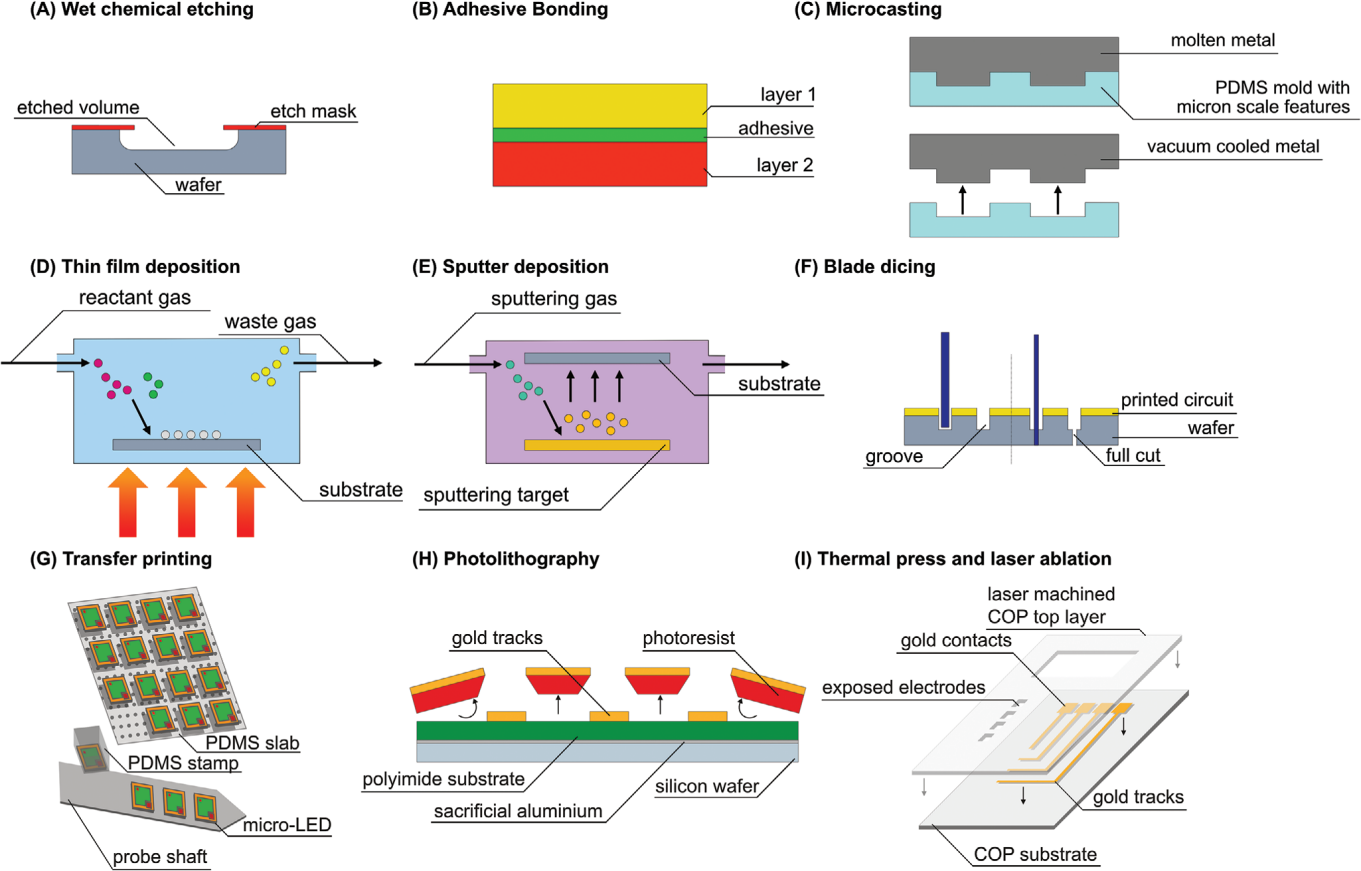
Various fabrication methods are explained above: A) wet etching, B) adhesive bonding, C) microcasting, D) thin film deposition, E) sputter deposition, F) blade dicing, G) transfer printing of micro‐LEDs (redrawn from^[^ ^332^ ^]^), H) simple fabrication using thermal lamination of gold on COP (redrawn from^[^ ^151^ ^]^), and I) traditional photolithography with gold deposition on polyimide.

The most common methods of fabrication for micron‐scale neural probes necessitate the use of nanofabrication facilities, employing techniques such as photolithography, thin film deposition, and various etching styles, among others.^[^ ^262^ ^]^ There are incredibly high standards imposed on medical implants before they are deemed safe for use in human tissue.^[^ ^185^ ^]^ As such, there is an emphasis on manufacturing techniques which will prevent device failure and ensure the longevity of the implant. This report provides a brief explanation of the nanofabrication techniques implemented in neural implants, as shown in **Figure** **7**.

### 6.1 Nanofabrication Methodology

When considering flexible polymer probes, often the first step is to deposit or solidify the substrate layer. This is often accomplished using spin coating and baking. Thin film deposition is a general term which refers to techniques including, but not limited to, chemical vapor deposition (CVD), which is an important step in the encapsulation of implantable devices in parylene‐C. Increasingly miniaturized transistors necessitate films which are typically 5 nm thick, reduced to the point where the results of CVD are not always repeatable.^[^ ^263^ ^]^

To create the electrodes, tracks, and contact pads, evaporation or sputter deposition is employed: this is a form of physical vapor deposition from which electrodes are detailed after lift‐off.^[^ ^109^, ^264^ ^]^ Interestingly, patterning may be achieved through microcasting: this generates features with micron‐scale dimensions.^[^ ^265^ ^]^ Wet etching allows for the removal of polymer layers, useful when exposing the electrodes so that they may make contact with the target tissue. This is also known as isotropic etching,^[^ ^266^ ^]^ and uses liquid chemicals to remove material; in dry‐etch processes, wafers are bombarded with ions to the same effect.^[^ ^109^, ^264^ ^]^ Adhesive bonding may be employed when there are several layers of polymer which must be bonded together and sealed. This technique improves the likelihood of the implant being biocompatible, and comprises of the adhesive hardening or solidifying to join together two surfaces, possibly using heat to facilitate the reaction.^[^ ^267^ ^]^ The increased time and expense required for adhesive bonding is offset by increased joint stiffness, fatigue strength, and the variety of materials which may be bonded together.^[^ ^268^ ^]^

When using silicon as a probe substrate, there must be some method of shaping a wafer into a thin probe shaft. Wafer dicing often produces fragile chips, typically with defects;^[^ ^185^ ^]^ despite this, it is employed as a simple and repeatable approach to achieve the probe shape. If the probe is especially long, with a reduced width, care must be taken to ensure the wafer does not have any defects which could cause fracture during implantation.

Transfer printing allows for stiff electronics to be fabricated first on a traditional substrate, and then transplanted to a more suitable (flexible, polymeric) substrate: this prevents any damage to the receiver substrate which is not well suited to nanofabrication techniques.^[^ ^269^ ^]^ Nanoscale manipulation has also been made possible through transfer printing techniques, with precision largely unmatched by techniques such as optoelectronic tweezers. PDMS ‘*μ*‐stamp[s]’ have been used to arrange nanowire lasers with a precision in the nanometer range.^[^ ^202^ ^]^ Interestingly, transfer printing appears to be a powerful and transferable method of manipulating micron scale electronics, allowing for independent manufacture of the component part required for a neural probe.

### 6.2 Successful Fabrication Protocols

Neural probes are typically produced in a cleanroom environment, and these traditional, relatively inexpensive techniques may be used to produce increased electrode areas:^[^ ^270^ ^]^ 50 × 50 µm^2^ recording electrodes, and a shaft width of 525 µm. First, the silicon substrate was spin‐coated with photoresist, which was then exposed to ultraviolet (UV) light through a mask and developed. An adhesion promoting layer of titanium was deposited using e‐beam, followed by sputter deposition of platinum to create the electrodes and connections. A second photolithography routine was carried out, with the addition of blade dicing in between the UV exposure and developing; the photoresist acts as a protective layer during dicing. Finally, silicon nitride was sputtered onto the device for encapsulation purposes. A range of testing showed that for even the smallest fabricated electrode, when the photolithography process was successful, the final impedance aligned with recommended impedances for neural probes.^[^ ^271^ ^]^

While parylene‐C is a common choice for polymer probes, the soft substrate can also be combined with a number of other techniques to improve the robustness and performance of the neural probe. A parylene substrate may be coated with a PVA/Poly(lactic‐*co*‐glycolic) acid (PVA/PLGA) mixture to provide the necessary strength for insertion.^[^ ^272^ ^]^ Notably, in the final fabrication step the device was spin coated with PEDOT:PSS, which is a conductive solution, before the top sacrificial layer of parylene was removed such that only the electrodes were coated. This served to decrease the impedance and increase the SNR.

Prioritizing simple fabrication, films of COP may be thermally pressed and laser machined to provide layers of substrate and encapsulation, as well as a desired probe shape.^[^ ^151^ ^]^ COP acts as a substrate for gold stimulation electrodes without the use of cleanroom photolithography. The miniaturization process was aided by UV laser machining, in which a laser with a reduced beamwidth yields smaller features. Gold was thermally laminated onto a COP substrate, which was covered over with a second COP encapsulation layer after the gold layer had been machined, leaving electrodes and connective lines behind. A CO_2_ laser was used to cut through the COP top layer and expose areas of interest on the conductor: mainly, the electrodes at the tip of the shaft and connections at the top. In the case of the COP thermally laminated probe, the minimum width was not comparable to mesh electronics, or even the state of the art in traditional probe shafts. This technique lacks the precision required to successfully target and record single unit potentials.

## 7 Implantation

**Figure 8:**
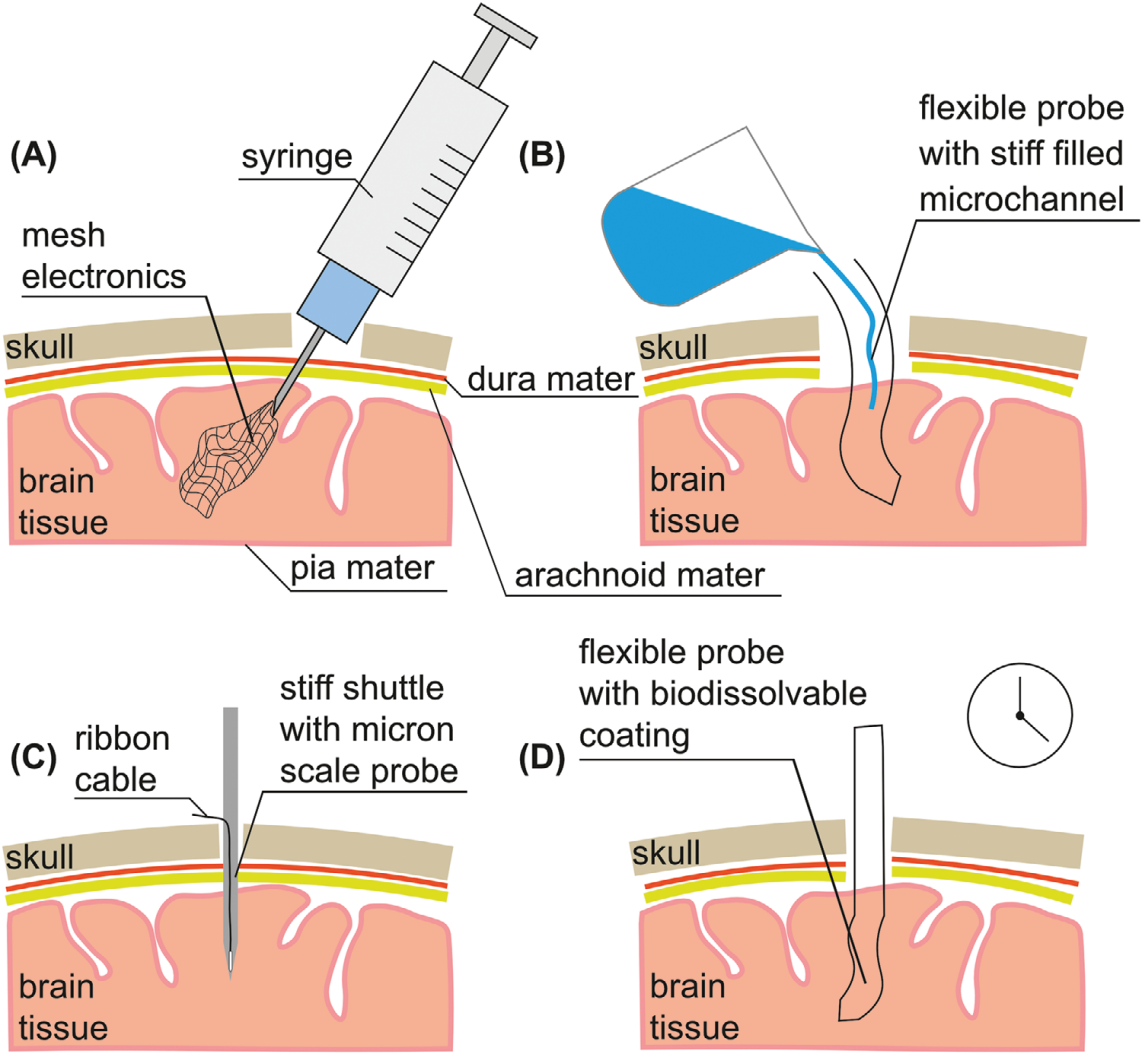
A) Syringe injectable mesh electronics. B) Microchannel in flexible probe filled with fluid to provide stiffness. C) Rigid shuttle and miniaturized probe with ribbon cable attached. D) Flexible probe coated in a biocompatible dissolvable material.

The method of implantation is dictated by the characteristics of the neural probe. While stiff probes require simpler surgeries, recent emphasis on flexible probes means that alternative implantation methods must be explored. Each mechanism of implantation is illustrated in **Figure** **8**, and it is clear that they each hold merit.

### 7.1 Surgical Techniques

With the great aspiration of implanting neural microelectrodes, stereotactic neurosurgery is a procedure commonly employed by neurosurgeons to find the finest strategies to reach the area of interest within the brain, minimizing any potential risks for vital structures. It commonly requires the use of advanced imaging techniques. The generation of high‐resolution 3D imaging data, to ensure the safe insertion of neural device according to a specific 3D coordinate system have been widely studied in animal models. In vivo imaging techniques, such as two‐photon microscopy is explored as a promising tool to provide 2D and 3D reconstruction of neurovascular networks with a subcellular resolution and allow the study of tissues responses as consequences of neural probe implantation.^[^ ^273^ ^]^ Moreover, this procedure is important to find the best routes for the target area and reduce vascular intracerebral edema that may lead to neuroinflammatory response and electrode defeat.^[^ ^273^ ^]^ Nowadays, there are still some limitations in using this technique for human brain surgical planning, and noninvasive neuroimaging methods, such as functional magnetic resonance imaging (fMRI), are commonly applied for specific epilepsy and tumor surgeries.^[^ ^274^ ^]^ Interestingly, the usefulness of real‐time two‐photon image recording has been recently demonstrated in living human ocular tissues, representing a great opportunity for in vivo neurological investigation in future.^[^ ^275^ ^]^

Among different surgery techniques, very significant is the development of computer‐driven system, such as robotic technology for stereotactic neurosurgery which provides the surgeon a high‐precision manipulation system, minimally invasive and accurate stereotactic approaches, and image‐guided procedures, reducing the risk of human errors and operating time.^[^ ^276^ ^]^

A very recent progress in the area of brain‐computer interface technology is the development of the so‐called “sewing machine” for the brain. The major goal of this research is the fine implantation of miniaturized flexible neuromorphic devices with minimal invasive tissues damage. The first application of this new approach was performed implanting recording electrodes in rat somatosensory cortex but further studies are needed to confirm its possible clinical application.^[^ ^277^ ^]^ Due to the very novel nature of this technology, Hanson et al. are yet to publish their work in a peer reviewed journal. Nevertheless, sewing machine approach will open the door to major knowledge of brain tissue and, in the near future, to the treatment of neurological conditions.

### 7.2 Insertion Forces

Compliant flexible neural probes exhibit higher mechanical compliance, and they show promise in enhancing the long‐term performance, comparing to traditionally stiff and needle‐like neural probes.^[^ ^41^, ^278^, ^279^, ^280^ ^]^ However, the main disadvantage of flexible designs is their challenging insertion procedure, for which numerous overcoming techniques have been proposed and demonstrated by different groups. Reliability of insertion is a major issue for flexible probes, and because of their much lower Young's Modulus than silicon‐based probes (e.g., Utah Array and Michigan), they are less stiff and less able to pierce the brain tissue.

Yielding/bending, rapture and buckling are mechanical failures that can occur during insertion. Buckling failure is more dependent on the probe geometry, while rapture and yield failures mainly depend on material properties.^[^ ^281^ ^]^ Critical buckling load (force) is important, because in practice, it can prevent insertion misalignment, breakage or tissue fracturing.^[^ ^100^ ^]^ Therefore, a precise and successful insertion requires a quantitative analysis of design parameters, such as probe shank parameter, length, and tip size/shape. Always there is a tradeoff between critical buckling force and probe shank diameter, i.e., thinner shanks are more susceptible to buckling failure because of their smaller critical buckling load. The insertion method, in which insertion speed and angle are varied, has a significant impact on the success rate. In the case of a thin silicon probe, altering the insertion protocol (reduced speed and increased insertion angle compared to the perpendicular) reduced the insertion force by a factor of six.^[^ ^282^ ^]^

In different studies on rat brain, insertion forces through the pia matter have been measured to be 0.5–2 mN, using different probe geometries and material.^[^ ^283^, ^284^ ^]^ Probe shank tip shape, size and sharpness can highly affect insertion force. Studies show that probe tips with opening angles of <20° can penetrate dura without dimpling, but for probe tips with opening angle of >40° difficult dura penetration has been experienced.^[^ ^285^, ^286^ ^]^ Various tapered probes have been investigated and pia penetration forces of 0.48–1.15 mN have been reported for different tip opening angles and shank diameter.^[^ ^283^ ^]^

### 7.3 Stiff Probes Implantation

One of the most well‐known neural probe design projects was undertaken in 1981 at Michigan University: the silicon‐based design which is now known as the Michigan probe. At its tip, a Michigan probe may be only 20 µm wide.^[^ ^287^ ^]^ While the rigidity of the probe would surely generate an immune response and further damage during micromotion, the silicon shaft is noted as being strong enough to pierce the dura mater. In an attempt to compromise between flexibility and precision, a rigid silicon probe can be combined with a flexible ribbon cable: this makes for an essentially “floating” probe which is not adversely affected by micromotions or connections to the skull.^[^ ^288^ ^]^ While an immune response will still be invoked by the stiff probe, in which encapsulation will increase the electrode impedance, it has been reported that recording is still possible after a number of weeks.

### 7.4 Shuttle

The specifics of neural probe insertion using a shuttle or insertion rod^[^ ^289^ ^]^ first involve the preparation of the skull, dura mater and pia mater. Once a burr hole has been made, the flexible probe is pushed down into the brain tissue, wrapping around the shuttle as it goes. When the insertion rod is removed, the flexible probe remains in place. Although this allows for the very precise placement of the probe, there are a number of disadvantages to consider: the removal of the shuttle causes further surgical trauma;^[^ ^272^ ^]^ an implantation lesion and further glial layer may be observed; and the surgery itself requires careful handling. A sharpened shuttle would be capable of piercing the dura mater and delivering probe arrays to the intended position in the brain.^[^ ^161^ ^]^ Maintaining the integrity of the dura mater is important for preventing inflammation and bleeding. After sharpening the tip of the shuttle in all three dimensions, and successfully implanting a probe through the dura, SUA has been recorded for 95 days. The use of nontraditional shuttles resulted in reduced bleeding during implantation and low insertion force,^[^ ^182^ ^]^ which is also impacted by the topography and size of the probe, as illustrated using microthread electrodes.^[^ ^290^ ^]^ Shuttle design will take cues from the most successful rigid probes, exploiting the best aspects of a rigid, temporary component and a flexible, chronically implanted probe.

### 7.5 Biodissolvable Coating

In order to temporarily stiffen a flexible probe, various bioresorbable coatings are explored in terms of their rigidity, biocompatibility, and time required for dissolution. Bioactive or biofunctionalized coatings have been discussed in Section 3.6. However, such a coating may also contribute to the mechanical characteristics of the probe in the short term. There are several benefits to a hybrid PVA/PLGA hybrid coating:^[^ ^272^ ^]^ both of these polymers have their individual advantages, although both are biodissolvable and often biocompatible. In Pas et al.,^[^ ^272^ ^]^ the body of the probe was coated with PVA, while the tip was coated with PLGA. The pairing of the readily dissolvable PVA with the hardier PLGA allows a sharp probe tip to be maintained throughout surgery and beyond if required. Silk is another example of an organic polymer which is incredibly strong and ordinarily biocompatible.^[^ ^291^ ^]^ Polyethylene glycol (PEG), which begins to degrade within minutes of insertion, is often used to stiffen the probe shaft.^[^ ^233^ ^]^ It has been certified as food‐safe by the FDA,^[^ ^292^ ^]^ and deemed biocompatible for medicine delivery in the central nervous system, with the immune response around the PEG hydrogel limited to a 50 µm radius.^[^ ^293^ ^]^

### 7.6 Microchannel

Microchannels in neural probes may be filled with stiff materials during insertion,^[^ ^294^ ^]^ which are removed once the probe is implanted, such that only the flexible probe remains. One such material is PEG, which is used as a base for many hydrogels. The microchannel may also be used for drug delivery after implantation, in order to mitigate the inflammatory response which prohibits chronic implantation.^[^ ^295^ ^]^ During recording, when flexibility is paramount, the channel could be filled with water.

### 7.7 Syringe

Since mesh electronics may be implanted using a syringe with diameter < 100 µm, trauma is reduced during initial insertion. However, since the mesh “unrolls and expands,” this technique lacks the precision of more traditional, larger, shuttles or rigid probes. When mesh electronics are compared to thin film electronics,^[^ ^296^ ^]^ the latter are not readily injectable, even with reduced dimensions compared to mesh electronics. There can be no doubt that the competing stiffness requirements of a neural probe during insertion and chronic implantation cannot be met without a hybrid approach. In the ideal case, the implantation mechanism is stiff, and the chronically implanted probe is soft and flexible. While the syringe used to implant mesh electronics appear to solve this problem, the syringe is a niche method which is only applicable to a fine mesh: even thin film electronics cannot be successfully implanted. Most notably, the syringe has the option of piercing the dura mater without further surgical trauma involved in trepanning and removal of the dura.

While mesh electronics are best suited to insertion via syringe, the novel Injectrode from Trevathan et al. is designed in such a way that the electrode is inextricable from its implantation method.^[^ ^297^ ^]^ Silver particles embedded in an uncured silicone elastomer matrix are injected around a nerve to cure inside the body, allowing the electrode to conform precisely to its target tissue. This innovative design is defined by the minimal invasiveness of the surgery. Once cured, the Injectrode has a Young's modulus of only 72.1 kPa, and as such significantly reduces the mechanical mismatch between probe and tissue.

### 7.8 Alternative and Custom Insertion Methods

The insertion methods discussed previously represent some of the most common techniques; however, lesser‐used methods have their own unique benefits. Two notable works involve using a metal rod or needle as temporary insertion aids, without adhering the polymer probe along the length of the rod.

By etching a hole into the tip of a polyimide probe, Zhang et al. created a simple system in which a needle could be threaded through this hole and adhered with a small amount of PEG.^[^ ^298^ ^]^ This could then be used to insert the polyimide probe before the PEG dissolved in the brain, after which the needle could be removed. After in vivo evaluation of this technique, local field potentials could be recorded from the CA3 region of the rat hippocampus for at least seven days.

Successful in vivo implantation was carried out by Richter et al.^[^ ^289^ ^]^ The inserted polyimide probe measured 20 µm × 350 µm × 1.5 cm and after the skull of the rat was opened, the flexible probe could be placed flat onto an “agarose cushion.” At this point, a tungsten rod was placed at the midpoint of the polyimide probe, and used to push the flexible shaft down into the brain tissue. After seven days, only a “mild tissue reaction” was observed.

Similarly, an insertion guide which is placed on the brain surface is employed to reduce the decrease the effective length of the probe.^[^ ^299^ ^]^ Fabricated using poly(methyl methacrylate) (PMMA), the insertion guide increased the maximum force which could be applied to the probe (before buckling) by more than a factor of 3. This was tested in vivo, with a 100% insertion rate when employing the guide versus only 38% success without.

PEG has also been employed to temporarily shorten the length of the flexible probe shaft,^[^ ^300^ ^]^ when poured over the top half (in the length direction) and cured. As such, the buckling force was measured in a brain phantom, and was significantly improved to 2.14 mN for a braced probe.

## 8 Energy Transfer and Harvesting

Wireless power transfer is an ideal approach to provide continuous power to the implant, especially when the power transfer or harvesting systems are miniaturized or biocompatible. Consequences of conventional powering techniques are numerous, beginning from the negative effects of repeated surgeries,^[^ ^301^ ^]^ severe foreign body response,^[^ ^12^, ^13^ ^]^ or even temperature issues surrounding the battery as an effect of the power consumption. Through the use of wireless power transmission techniques, the long‐term viability of the implant is improved enormously, in terms of integration with the brain tissue reducing the adverse response of the tissue against the materials and big shapes that commonly are used in battery‐based architectures. For that reason, this is the most common choice in the design of novel bioimplantable devices, and in particular, for brain stimulation as reported by other authors.^[^ ^59^, ^105^, ^302^ ^]^ Besides, wired power and data transfer requires “tether[ing]” of the test subject to an external device, which will induce micromotions.^[^ ^303^ ^]^ Requirements for stimulation and recording are different in terms of device design.^[^ ^59^, ^105^, ^302^ ^]^ While for stimulation the main design constraint is focused on preventing any brain tissue damage; in neural recording, the main limitation is the apparent recording site impedance which changes over time as a result of the cellular growth of brain tissue around the neural probe. This increasing number of cells around the neural probe affect the efficiency and the quality of the neural recordings as an effect of a higher impedance that reduces how the signals are recorded. These sorts of limitations must be taken into consideration in the design phase and during implantation period since the voltage requirements vary with the time. Communication limitations and implant size restrictions are not generally dependent on stimulation or recording architectures.

Despite focusing on the benefits of wireless power for implantable devices, it is important to briefly mention the aspects of wired devices which may impede the natural movement of test animals, or perhaps dictate the design of the probe as it connects outside the skull. Headstages are employed both in wireless and wired systems, although a wired system must also include an external connector. Incorporating recording electronics and signal enhancement,^[^ ^304^ ^]^ Limnuson et al. combine a straightforward wired design with a rotating commutator to allow for rats to move unimpeded. The headstage may also be used to protect the implant inside a rodent brain, preventing trauma which will damage the neural probe and the rodent itself: alleviating this risk means that rats may be pair‐housed in a more natural, stimulating environment.^[^ ^305^ ^]^

The final component of the implantable system is the connector which allows a cable to interface with an external computer. Three common options for these connectors include zero insertion force (ZIF) connectors,^[^ ^177^, ^306^, ^307^ ^]^ which may be combined with polymer probes; or rigid alternatives such as Omnetics^[^ ^110^, ^233^ ^]^ or Samtec^[^ ^308^, ^309^ ^]^ connectors.

The miniaturization of recording electronics, combined with light and potentially flexible wireless power transfer systems, mean that the future of implantable probes lies in completely untethered implants.

**Figure 9:**
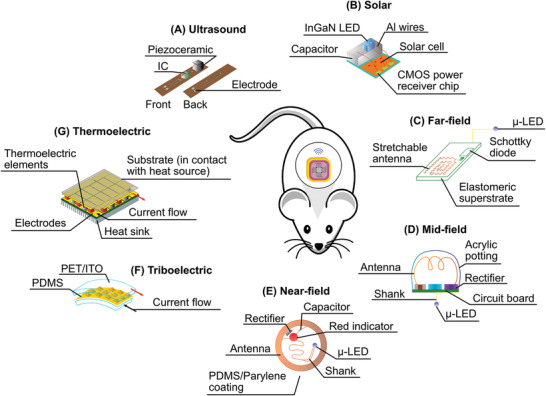
A) Piezoelectric energy harvester. B) Flexible subdermal solar cell. C) Far‐field electromagnetic coupling. D) Mid‐field electromagnetic coupling. E) Near‐field electromagnetic coupling. F) Triboelectric energy harvester. G) Thermoelectric energy harvester. A–E) Adapted.^[^ ^303^ ^]^ F) Adapted.^[^ ^368^ ^]^ G) Adapted.^[^ ^326^ ^]^

However, a key challenge lies in the miniaturization of the system:^[^ ^301^ ^]^ without a reduced implant size, power transfer may only be realized through a thin layer of tissue. Advancements in various types of technologies for power transfer (ultrasonic and electromagnetic) and energy harvesting (photovoltaic, thermoelectric, triboelectric) are discussed below in detail, as illustrated in **Figure** **9**. The characteristics of these power transfer systems are highlighted in **Table** **4**.

**Table 4:** Characteristics of wireless power transfer and energy harvesting technologies. The most important aspect of such a system involves balancing the size of the implant with its power output

Power transfer system	Generates	Biocompatibility	Experiment length	Experiment type	Flexibility	Size	Reference
Ultrasonic	Harvests maximum of 5 V_pp_	Encapsulated with parylene‐C	N/A	In vitro	Flexible PCB	3.8 mm × 0.84 mm × 0.75 mm	^[^ ^391^ ^]^
Ultrasonic	22.25% electromechanical coupling	Encapsulated with PDMS	N/A	Tested in water	Yes	16 elements measuring 1 mm × 1 mm × 100 µm	^[^ ^392^ ^]^
Triboelectric, ultrasonic	2.4 V × 156 µA	N/A	N/A	Ex vivo	Flexible PCB and polymer layers	4 cm × 4 cm	^[^ ^393^ ^]^
Electromagnetic	Coupling 500 mW, receives 200 µW in the brain	Far below SAR limit for 500 mW coupling	N/A	In vivo	Rigid patterned metal	2 mm diameter, 3.5 mm height, weighing 70 mg	^[^ ^59^ ^]^
Electromagnetic	19 mW	Planned for future work	N/A	Computer Model, Phantom	No	0.9 mm^3^	^[^ ^317^ ^]^
Photovoltaic	90 µW minimum	Planned for future research	34 min	Ex vivo	Yes	64 × 37 mm	^[^ ^394^ ^]^
Photovoltaic	647 µW	Encapsulated in PDMS	5 weeks	In vivo	Yes	760 µm × 760 µm × 5.7 µm, plus encapsulation layers	^[^ ^320^ ^]^
Photovoltaic	60 µW	Yes, tested in vitro	3 days	In vivo	N/A	390 µm × 410 µm × 1.5 µm	^[^ ^395^ ^]^
Triboelectric	4 V	Encapsulated with PVA or PLGA	9 weeks	In vivo	Yes, polymer‐based	2 × 3 cm	^[^ ^322^ ^]^
Thermoelectric	645 µW	Biocompatible insulator used	20 min	In vivo	N/A	Surface area 0.83 cm^2^	^[^ ^329^ ^]^
Thermoelectric	25 mV	Requires encapsulation	>260 s	In vitro, in vivo	No	10 mm × 10 mm × 3.9 mm	^[^ ^325^ ^]^

### 8.1 Ultrasound

While electromagnetic coupling may be used to power medical implants, relatively long wavelengths and high losses within the body mean that ultrasound is a contender for a more efficient, miniaturized, wireless power transfer system. The benefits of ultrasonic systems are identified in terms of their compatibility with human tissue.^[^ ^310^ ^]^ First, the safe exposure level for ultrasonic power is significantly higher than that of radio frequency (ultrasonic, 720 mW cm^−1^ and radio frequency (RF) 10 mW cm^−1^, respectively), while the power losses associated with ultrasonic transmission through tissue are comparatively lower than RF losses.

Frequency is an important issue in implantable wireless devices: acoustic waves have much lower velocity compared to electromagnetic waves,^[^ ^311^ ^]^ with wavelengths in the order of millimeters, and low frequencies. As such, ultrasonically powered systems may be miniaturized further than the antennas required for electromagnetic transfer, without compromising on efficiency.^[^ ^312^ ^]^ The importance of miniaturized device footprint is highlighted through the careful development of a 0.8 mm^3^ neural implant. Ultrasonic power is also not so dependent on the alignment of the device, compared to the alignment of antennas in an electromagnetic system; this will make for more uniform power delivery between patients.

A standout technology utilizing ultrasound power transfer for both EMG and EEG recording is neural dust, which can also perform wireless communication with a “vanishingly small”^[^ ^313^ ^]^ piezocrystal implant and external Bluetooth module. Seo et al. have published a well‐rounded body of work on their neural dust technology, from MATLAB simulations^[^ ^314^ ^]^ to in vivo validation^[^ ^315^ ^]^ and a wearable system for untethered rodent experiments.^[^ ^316^ ^]^

### 8.2 Electromagnetic

Electromagnetic wireless power transfer relies on the resonance phenomenon. It makes use of two antennas, defined as conductive structures that are resonant at a certain frequency. There are different shapes, geometries and materials where the antennas can be implemented. Since size matters for bioimplantable devices, higher frequencies are often chosen, reducing the sizes of the antennas. However, receiver and transmitter antennas can be in different geometries if and only if they are resonant at the same frequency. The electromagnetic wave goes through the tissue from transmitter to receiver antenna. Depending on the distance from the two antennas, the typologies are named near‐field, mid‐field, or far‐field and they present different characteristics and limitations.

Some of the difficulties introduced by near‐field coupling include the fact that brain tissue is lossy, near‐field transfer undergoes exponential decay, and there is weak coupling between transmit and receive coils when they are not properly aligned.^[^ ^59^ ^]^ Despite this, innovative antenna design yields reduced footprints and high efficiency: for example, successful antenna designs may have an antenna diameter of less than 1 mm.^[^ ^317^ ^]^

Mid‐field coupling is used to construct a power transfer system which would be equally effective no matter where it was implanted in the body.^[^ ^59^ ^]^ The whole device prepared for implantation was only 2 mm in diameter and 3.5 mm in height and can deliver 195 µW to the heart and 200 µW to the brain when the source output is 500 mW. The most favorable regime is mid‐field for power transfer, due to the observed efficiency at the selected ideal frequency ($\frac{1}{d^{3}}$), where in this case *d* refers to the distance between transmit and receive antennas.^[^ ^318^ ^]^

The far‐field approach has several benefits: most notably, the reduced coil diameter with increased operational frequency. A 3 × 3 mm antenna operating in the far‐field is capable of powering an implant at a distance of 20 cm.^[^ ^39^ ^]^ While the increased losses associated with far‐field power transfer may be compensated by increasing the transmitted power,^[^ ^301^ ^]^ any solution must take into account specific absorption rate (SAR) limits for safe operation.^[^ ^39^, ^318^ ^]^

Electromagnetic wireless power transmission techniques are not exempt from risk. One of the most studied influences of electromagnetic waves on the human tissue is the SAR which was addressed before. However, this issue can be relatively easy to control as it depends on physical parameters that can be modelled.

### 8.3 Photovoltaic

Implanted solar cells are smaller, lighter, and more flexible than battery implants. These absorb light in the near‐infrared and visible regions, incident on the cell even through layers of human skin. Cells may absorb both natural and artificial light, illustrating the success of photovoltaic cells for low‐powered implants.^[^ ^319^ ^]^ In general, the power generated by a solar cell during everyday activities is sufficient to power a pacemaker, without any particular efforts made to increase the light incident on the implant. Existing implanted solar cells are typically made from silicon, which have been reported to generate over 600 µW of power.^[^ ^320^ ^]^ However, its stiffness causes damage to the surrounding tissues and skin. Two challenges presented by solar implants are the need for thin, flexible cells, as well as improved biocompatibility of the cell substrate to remove further encapsulation.^[^ ^312^, ^320^ ^]^ Alternatively, bioinert and bendable encapsulation materials such as those used for neural probes are also effective.

### 8.4 Triboelectric

The triboelectric effect, which converts biomechanical energy into useable electrical energy, has been employed to power wearables on the surface of the skin,^[^ ^107^ ^]^ and more recently, it has been explored as a power harvesting method for biomedical implants.^[^ ^321^ ^]^ Using materials such as PLGA (see Section 7.3, Biodissolvable Coating),^[^ ^272^ ^]^ patterned layers of polymer create frictional surfaces. When these layers are forced together and then detached, this induces a voltage which can be used by to stimulate cells. A stimulator may be made using familiar materials^[^ ^322^ ^]^ such as PDMS and polyimide. PDMS has also been used as a patterned layer in conjunction with an ionogel^[^ ^323^ ^]^ to create a biocompatible and conformable device, with a footprint of 3 cm^2^ and a thickness of 1.2 mm. Movement on the surface of the skin, or even on cardiac tissue, is rarely sufficient to power a neural implant. Coupling a triboelectric energy harvester with external ultrasound transmission^[^ ^311^ ^]^ to create a “vibrating and implantable triboelectric generator (VI‐TEG),” it is possible to charge a battery of the sort implanted alongside a pacemaker.

### 8.5 Thermoelectric

The low efficiency of thermoelectric generators (TEGs) is offset by their high reliability, which is crucial in chronic implantation cases. µ‐TEGs have been used to power biomedical electronics for the heart, ears, and brain,^[^ ^324^ ^]^ with dimensions in the order of 1 cm. The temperature differential between the human tissue and the device generates a current in the elements of a TEG, which is ideally placed close to the skin. Using a sample of porcine skin and fat, a 3.3 mV output was achieved with an implanted TEG. This was subsequently improved by cooling the skin to increase the gradient between the core and surface temperatures of the body, which provided an output voltage of 6 mV.^[^ ^325^ ^]^ Flexible materials have been explored for wearable TEGs,^[^ ^326^ ^]^ since improved conformability to the heat surface will also improve the heat transfer capability of the device. Once such flexible material is PEDOT:PSS, which boasts excellent biocompatibility,^[^ ^327^ ^]^ and so would be suitable for implantable TEG applications. It has also been suggested that the device is placed close to an artery, for example the carotid artery. Through natural heat loss mechanisms, up to 32.3 W of heat is lost there, although this is dependent on the flow rate as the carotid supplies the brain.^[^ ^328^ ^]^ During in vivo testing in a rat brain, careful design of a lightweight TEG, with a surface area less than 1 cm^2^ reportedly yields 645 µW of power.^[^ ^329^ ^]^

## 9 Design Recommendations

While the key aspects of neural implant design have been discussed in this progress report, it is important to summarize the conditions in which a device is most likely to succeed. Although previous research has yielded guidelines for the ideal implant, limitations in fabrication, surgical insertion, and material science are only some of the challenges which must be overcome to reach this specification.

### 9.1 Mechanical Flexibility

As highlighted in Section 4, the closer the Young's modulus of the shaft material to brain tissue, the more successfully the implant integrates with the host tissue: rigid substrates such as silicon are likely to cause increased damage during chronic implantation due to micromotion. With the aim of encouraging neuronal growth, the substrate should be as flexible as possible: neurons can grow three times the number of branches^[^ ^94^ ^]^ when adhered to a soft substrate when compared to a rigid one. A flexible substrate acts to reduce the interfacial force on the implant,^[^ ^182^ ^]^ preferably until it is equivalent to the forces which occur during natural processes such as inflammation and the growth of blood vessels.^[^ ^330^ ^]^ Despite the importance of substrate flexibility, there is a limit to the efficacy of polymer probes, when no other techniques are employed to improve the chronic implantation case. When comparing silicon with polyimide and off‐stoichiometry thiol‐enes‐epoxy (OSTE+), the FBR is greatly reduced in the case of polyimide versus silicon. However, while OSTE+ has a much lower Young's modulus even than polyimide (6 MPa vs 1.5 GPa), the presence of immune markers is not significantly changed by the use of OSTE+.^[^ ^63^ ^]^

### 9.2 Stretchability

On the theme of micromotion and tissue damage, the selected substrate should be stretchable to accommodate for any force incident on it, and the motion involved in free movement of the host, breathing, or the changes associated with blood flow. Reported values for micromotion vary; however, micromotion of at least 30 µm should be expected for brain tissue.^[^ ^97^ ^]^ Various methods for increasing the stretchability of the probe include creating slits in the polymer substrate,^[^ ^246^ ^]^ the addition of a plasticizer,^[^ ^327^ ^]^ or patterning a prestrained polymer to ensure the metallic tracks remain unbroken even after the substrate is stretched to twice its original length.^[^ ^331^ ^]^ Silicon substrates are ubiquitous in the fabrication of micron‐ and nanoscale devices, with extensively developed protocols; on the other hand, devices are not routinely fabricated on a flexible substrate, instead diced and etched for transfer between the original rigid substrate and the polymer layer. Specialized techniques such as transfer printing^[^ ^332^ ^]^ are integral to this multistage process.

The key difference between substrates such as polyimide versus PDMS is that while both exhibit flexibility, the stretchability of PDMS is inherent, whereas polyimide can be made stretchable through techniques such as kirigami.^[^ ^333^ ^]^ Both characteristics are key to biomimetic electronics. However, stretchable electronics are viewed as a further development towards truly conformable devices and circuits, and a “significant departure”^[^ ^334^ ^]^ from their solely flexible counterparts. When adhered to the skin, for example, a flexible substrate cannot wrinkle naturally as skin would:^[^ ^335^ ^]^ a stretchable substrate conforms completely to the surface it is adhered to. Finally, stretchable electronics are better suited to rounded surfaces when compared to simply flexible electronics.^[^ ^336^ ^]^

### 9.3 Miniaturization and Scalability

The neural implant topologies which are most successful at preventing prolonged immune response are miniaturized such that the feature size is of the order of the target cells.^[^ ^182^ ^]^ For example, mesh electronics successfully avoid invoking immune action, and were designed with “cellular or subcellular dimensions.”^[^ ^337^ ^]^ Standard photolithography is generally used for feature sizes greater than 1 µm; however, novel PDMS mask molds have illustrated features of the order of hundreds of nanometers.^[^ ^338^ ^]^ Electron‐beam lithography, which is both more expensive and time consuming, has nevertheless been reported to yield features smaller than 4 nm with great effort.^[^ ^339^ ^]^

### 9.4 Untethered

Typically, an implantable probe shaft will either be connected to a platform which incorporates electronics necessary for recording and stimulation or will be connected for wired communication. This contributes to tissue damage during chronic implantation: specifically, increased scarring and breach in the BBB.^[^ ^340^ ^]^ When comparing a floating electrode with one which is connected by wire, sealed with elastomer and linked to a connector secured to the skull, there is obvious benefit to the untethered electrode. The immune response was not merely increased in the tethered electrode: up to four weeks after implantation, the response to the secured electrode worsened, while the tissue around the floating electrode began to heal.^[^ ^103^ ^]^ The mechanism by which the electrode is anchored will impact the FBR, but not the density of neurons.^[^ ^341^ ^]^ During animal experiments, “physical tethers” will hamper natural behavior:^[^ ^105^ ^]^ this is significant when researching the neurological effects of an implant, rather than merely the immunological effects. It is important to note that wireless communication and power, while removing the need for bulky tethers, will in turn necessitate electronics for, e.g., rectification, voltage regulation and tuning.^[^ ^342^ ^]^

### 9.5 Biocompatibility

Section 4 highlights the characteristics which must be considered when choosing a polymer: chief among these is biocompatibility. This term denotes materials which have negligible negative effects on the body, and may also encourage tissue performance.^[^ ^343^ ^]^ While traditional neural probes allow the electrode tracks to be exposed to brain tissue, both synthetic and organic polymer encapsulation is used in modern probes to prevent corrosion and cytotoxicity. Implantable metals such as platinum or gold should be encapsulated when possible, and only exposed when contact is required for recording or stimulation. Probe failure, including delamination, should be prevented to mitigate toxicity to cells.

### 9.6 Open Volume

A macroporous,^[^ ^337^ ^]^ open design encourages neuronal penetration and successful integration of the implanted device. Decreased surface area helps to mitigate immune cell activation, damage to the BBB, and tissue death.^[^ ^87^ ^]^ It is important to note that even when compared to a shaft with identical dimensions, the volume of tissue surrounding the open implant showed marked improvement in tissue penetration. Material scaffolds in the field of neural implants are inspired by the ECM, which is a 3D mesh structure comprised of nanometer‐scale protein fibers.^[^ ^116^ ^]^ Mesh electronics, at the single‐micron scale, promote cell growth.

### 9.7 Reduced Impedance

Single unit activity recording requires both increased specificity, and decreased impedance.^[^ ^344^ ^]^ When incorporated with a “neurosensing system” the impedance of the probe must be carefully matched to the system to prevent electrical losses.^[^ ^345^ ^]^ Efforts may be made to reduce the electrode impedance, at the expense of the surface area, or by selecting conductors with lower impedance for the same surface area. Avenues to increase the electrode surface area without sacrificing the probe footprint include creating trenches, exposing more of the metal when compared to a flat, flawless surface.^[^ ^346^ ^]^ Alternatively, coatings such as PEDOT:PSS may be used to reduce electrode impedance by up to a factor of 10.^[^ ^347^ ^]^ Electrodeposited platinum‐iridium coating also serves to decrease the impedance of microelectrodes by a factor of 5, and improve the SNR.^[^ ^345^, ^348^ ^]^

### 9.8 Temperature

Electronic stimulation of neural tissue is accompanied by a change in temperature, much in the same way brain regions employed during natural behavior will also undergo a temperature increase. This is due to the dissipation of certain amount of electrical power, and the rise temperature over a safe threshold may be harmful for the tissue function. A maximum increase of 1 °C is acceptable when seeking to prevent tissue damage.^[^ ^44^ ^]^ This is a particulate concern for optogenetic stimulation, which requires the use of LEDs. Unsurprisingly, the temperature increase is correlated to the root mean square (RMS) of the stimulation voltage during DBS.^[^ ^349^ ^]^ Aside from temperature issues during normal operation, patients are at risk during MRI scans,^[^ ^350^ ^]^ which may be necessary at some point in their treatment for neurological disorders. Several steps should be taken by clinicians to remove risk to the patient: the use of a transmit‐head‐receive coil, switching the stimulator amplitude to zero, and switching it off.^[^ ^351^ ^]^

From a surgical standpoint, extensive review and experiment from Shoffstall et al.^[^ ^352^ ^]^ indicates that the temperature increases in brain tissue caused by drilling during craniotomy is significant enough in a rat brain that it will impact the neuroinflammatory response. Their findings indicate that saline irrigation is insufficient to prevent an increase in BBB permeability. Best practice for surgeons is to avoid aggressive drilling above 1000 rpm, and to introduce pulsed drilling to allow some time for heat to dissipate through the air, bone, and brain tissue. The maximum observed temperature increase was 21 °C, via an infrared camera.

### 9.9 Scar Tissue

The displacement of recording or stimulation electrodes from the main shaft of a neural implant aims to place the electrodes outside of the volume of tissue which is scarred, dying, or undergoing an immune response.^[^ ^353^ ^]^ This is also termed the “kill zone,”^[^ ^354^ ^]^ and can extend over 20 µm from the implant surface.^[^ ^355^ ^]^ Satellite stimulation sites on aggressively miniaturized “whiskers” extend into healthy tissue, beyond this region.^[^ ^353^, ^356^ ^]^ By carefully pacing the electrodes outside of the area of increased scarring (and by extension impedance, which will impact the selectivity of the probe), healthy neurons may be recorded, even after the FBR has encapsulated the main shaft. Perforations in the BBB cause particles up to 500 nm in size to amass in this volume of compromised cells:^[^ ^357^ ^]^ the molecules which leak through the barrier contribute further to neuronal degradation. The majority of the tissue damage caused during probe insertion may be tuned by altering the tip design. If the tip is both “ultrasharp” and “smooth,”^[^ ^286^ ^]^ it will slice through tissues with minimal resistance, and as such dimpling, tearing and stretching will be reduced. The goal is to reduce bleeding and ion leakage from cells which will be exacerbated by a blunt probe tip. Employing a “chisel‐point” tip, the kill zone is reduced to below 10 µm.^[^ ^353^ ^]^

### 9.10 Modulated Stiffness

A neural implant must be sufficiently stiff to undergo insertion without buckling or breaking, as well as facilitating precise placement of the electrodes. By contrast, the implant must also be flexible, with a Young's modulus which ideally approaches that of brain tissue.^[^ ^358^ ^]^ This may be achieved using a bioresorbable coating, which will dissolve harmlessly on contact with the fluids in the brain,^[^ ^272^ ^]^ a temporary shuttle, which is removed after the insertion procedure,^[^ ^161^ ^]^ or by introducing a mechanically adaptive substrate material which softens when implanted inside the brain.^[^ ^213^, ^214^ ^]^ Allowing a rigid implant to remain in the brain invites increased damage from micromotion. Significant brain shift is also observed in the course of implantation surgery. The insertion site must be carefully chosen to account for the movement of the brain up to two weeks after the procedure, during which time the probe leads typically deform into a “question mark” shape.^[^ ^359^ ^]^ During this brain shift, a rigid probe will not conform to the displacement of the soft tissue, instead causing further damage.

## 10 Conclusion and Future Outlook

In recent years, neural devices have shifted from stiff, wired implants, such as the Michigan probe, towards miniaturized, flexible and novel geometries to encourage successful chronic recording and stimulation. Accommodating the characteristics of neural tissues, careful design, fabrication, and implantation methods have created a generation of devices which show promising results in animal experiments. Specifically, approaches such as the NeuE and mesh electronics mimic the soft tissue, integrating with cells. In order to achieve this, biocompatible, flexible substrates, and encapsulation materials are employed. While the Young's modulus of these materials is typically order of magnitude lower than that of silicon, they still do not approach that of the brain. While these substrates are typically suitable to standard cleanroom patterning and etching, techniques such as transfer printing may be employed to improve the performance of the device. This versatility allows for the implementation of various stimulation methods: most recently, optogenetic probes with *μ*‐LEDS. Finally, in order to realize truly implantable devices, wireless power transfer is crucial to prevent repeated surgeries, disturbing the device and its surrounding tissues, and move past the reliance on battery powered medical implants. With daily advances in research, there is potential for these novel, sophisticated implants to be implemented for clinical application, to treat patients with neurological diseases, and provide better alternatives for the future.

## Conflict of Interest

The authors declare no conflict of interest.

## Supporting information

Supporting Information
